# A study on the non-contact measurement of sunflower disk inclination and its application to accurate phenotypic analysis

**DOI:** 10.3389/fpls.2025.1614898

**Published:** 2025-08-06

**Authors:** Qiang Wang, Kaixuan Li, Zihao Gao, Xinyuan Wei, Yaoyu Li, Yangcheng Lv, Wuping Zhang

**Affiliations:** College of Software, Shanxi Agricultural University, Taigu, China

**Keywords:** sunflower disk inclination angle, YOLO11-seg, precision agriculture, geometric analysis, intelligent harvesting

## Abstract

The tilt angle of sunflower flower heads is an important phenotypic characteristic that influences their growth and development, as well as the efficiency of mechanised harvesting in precision agriculture. Addressing the issues of low accuracy, high cost, and the risk of plant damage associated with traditional manual measurement methods, this study proposes a non-contact measurement method combining deep learning and geometric analysis to achieve precise measurement of sunflower flower head tilt angles. The specific method involves optimising the lightweight YOLO11-seg model to enhance instance segmentation performance for sunflower flower heads and stems (compared to the initial YOLO11 model, recall rate improved by 3.7%, mAP50 improved by 1.8%, a reduction of 0.29M parameters, and a decrease in computational load of 0.5 GFLOPs), and extracting the surface contour of the flower head and the centreline contour of the stem based on the mask map output by the model. After achieving precise region segmentation through image processing, the geometric analysis module performs elliptical fitting on the flower head contour to obtain the main axis direction, performs curve fitting on the stem contour, and selects the tangent direction at the intersection point of the flower head. The angle between the two is calculated as the tilt angle of the flower head. In the measurement experiment, 220 images were used for testing, with manual protractor measurement results as the reference. The algorithm achieved a measurement accuracy of RMSE = 2.93°, MAE = 2.43°, and R^2^ = 0.94. The results indicate that this method significantly improves measurement efficiency and operational convenience while maintaining accuracy. The system does not require contact with the plant, demonstrating good accuracy, adaptability, and practicality. The tilt angle information obtained is of great significance for path planning of harvesting robots, adjustment of gripping postures, and positioning control of end-effectors, and can serve as a key perception module in the automation process of sunflower flower head placement and drying operations in precision agriculture.

## 1 Introduction

Sunflowers are one of the world’s most important economic crops (Tursunov et al., 2023), widely used for vegetable oil extraction and seed consumption. Their seeds are rich in unsaturated fatty acids and protein, making them an important source of nutrition for a healthy diet (Adeleke et al., 2020). According to data from the Food and Agriculture Organization of the United Nations, sunflower oil is one of the world’s major sources of vegetable oil (Pilorgé et al., 2020). Global oil consumption is approximately 85 million tons, with vegetable oils accounting for 75% of this total, and sunflower oil holds a leading position within this category (Ali et al., 2020). Due to increasing consumer demand for oil content and seed quality, the spatial orientation of flower heads directly impacts product characteristics and economic value. Especially in export-oriented production systems, the orientation of flower heads is increasingly becoming a key criterion for evaluating “product consistency” and “oil stability.” Therefore, sunflower growth directly impacts vegetable oil safety and agricultural sustainability (Puttha et al., 2023). To address the pressure on grain and oil demand caused by population growth, increasing sunflower yields, improving quality, and enhancing stress tolerance have become core tasks in crop breeding and agricultural production.

To achieve the aforementioned objectives, the precise acquisition of crop phenotypes has become a critical step. Phenotypic analysis provides essential information on crop morphology, physiology, and developmental status, making it an indispensable tool for variety improvement, agronomic optimization, and high-throughput breeding (Centorame et al., 2024). However, existing research on sunflower phenotyping has primarily focused on traits such as plant height and leaf area index. While some studies have examined geometric features like flower head diameter (Takács et al., 2022), analyses of flower head tilt angle—a geometric feature—remain relatively limited. Sunflower phenotyping plays a critical role in enhancing crop yield, improving quality, and strengthening stress tolerance. For example, traits such as plant height, stem thickness, leaf area, flower head diameter, and tilt angle not only reflect plant growth status but are also closely related to final grain filling, seed yield, and oil accumulation (Sala et al., 2012). Therefore, how to efficiently and accurately measure flower head tilt angle has become a technical bottleneck constraining intelligent breeding and precision management.

The disk inclination angle, as one of the key morphological indicators (Ćuk et al., 2020), influences the extent to which the disk absorbs light and its utilization efficiency. In sunflower seeds, the seed kernel accounts for approximately 70% of the total seed weight, containing about 55% oil. Therefore, as an important spatial morphological characteristic, the flower disk inclination angle also affects oil accumulation levels and quality performance (Grompone, 2005). Research has found that east-facing flower heads have an average seed weight increase of 11.2% compared to west-facing flower heads, with more fully developed seeds, indicating significant advantages in pollination efficiency and seed development (Takács et al., 2022). Recent studies have shown that the disk inclination angle directly impacts sunflower oil production and seed commercial value. On one hand, an appropriate inclination angle enhances light utilization efficiency, promotes uniform kernel filling, improves seed plumpness, and thereby increases oil accumulation and quality stability (López Pereira et al., 2017). On the other hand, the reasonable control of the tilt angle has a significant impact on seed shedding rate, damage rate, and the process of sunflower head drying during mechanized harvesting. This is because different tilt angles affect the path planning, force and angle control mechanisms, and gripping angle design of harvesting robot arms, thereby influencing harvesting efficiency and seed damage rate, which directly relates to the integrity and market value of commercial seeds. If the tilt angle is estimated inaccurately, the end effector may not align properly with the surface of the flower head, resulting in incorrect contact direction, unstable gripping, and even damage to crops during robot operation.Research indicates that if harvesting begins at full seed maturity, seed loss increases by approximately 2 times on the 5th day and by 10–12 times on the 15th day (Stepanenko et al., 2022). This underscores the necessity of precise control over harvesting timing.

Current sunflower phenotyping methods primarily rely on manual measurements, including rulers, protractors, angle meters, and other handheld devices, to measure plant height, stem diameter, leaf width, flower head diameter, and inclination angle (DeValk et al., 2024). Although these methods offer simplicity and practicality, they have significant limitations, such as time-consuming and labor-intensive processes, high costs, substantial human error, poor reproducibility, and potential damage to plants during repeated measurements, particularly in terms of efficient data collection and real-time feedback.

Additionally, the measurement accuracy of traditional baseline models fails to meet the demands of precision agriculture and crop management (Monteiro et al., 2021). Meanwhile, with the development of non-contact technologies such as remote sensing, image processing, and artificial intelligence, crop phenotyping analysis is gradually shifting toward automation and digitization (Zhang and Zhang, 2018). Fieuzal and Baup utilized multi-temporal optical and SAR satellite data to estimate the leaf area index and crop height of sunflowers, achieving large-scale, high-precision crop phenotyping monitoring (Fieuzal and Baup, 2016). Sunoj et al. employed digital image processing techniques to measure sunflower inflorescence dimensions, achieving high measurement accuracy and semi-automated results (Sunoj et al., 2018). Although these studies have significantly improved measurement efficiency and reduced manual intervention, sensor-based systems still face major challenges in practical applications, including high costs, complex maintenance requirements, and limited adaptability to field conditions (Williams, 2019). In particular, sensor maintenance can account for up to 80% of the total deployment cost, highlighting the significant economic burden associated with sensor-based solutions (Yu et al., 2020). By employing image recognition methods, equipment and maintenance costs can be reduced, deployment flexibility significantly enhanced, and the technology’s scalability improved (Jia, 2025).

Meanwhile, deep learning-based image recognition and semantic segmentation technologies (such as Mask R-CNN and U-Net) have been applied to plant region identification and target detection, enabling automatic phenotyping in crops like wheat and corn (Alom et al., 2019; Huamao et al., 2023). However, these models still have significant limitations when it comes to spatial pose estimation. These models have low processing efficiency for large-sized, high-resolution images, consume significant memory (Liu et al., 2021), and their inference speed fails to meet the demands of field operations; Mask R-CNN experiences reduced segmentation accuracy under uneven lighting and complex background conditions, and also faces issues of error accumulation in the extraction of inclined boundaries (Hou and Jing, 2024). Zhang et al.compared the performance of U-Net, YOLOv3, Mask R-CNN, and an improved Mask R-CNN in the mixed forest canopy segmentation task. The results showed that U-Net and standard Mask R-CNN exhibited significant errors in boundary identification in complex structural regions, with Kappa coefficients of 0.70 and 0.76, and accuracy rates of 81.14% and 89.72% on the test set. The study noted that these models tend to produce inaccurate segmentation in areas with high canopy density and at target boundaries, which affects the precise extraction of boundary contours (Zhang et al., 2022). Furthermore, research has demonstrated that the YOLO series of object detection models show significant potential in crop angle phenotyping analysis. For example, He et al. (2024) proposed an improved YOLOv5 model called Swin-Roleaf, which combines the Swin Transformer with an angle classification mechanism called Circular Smooth Label (CSL). This method enables high-throughput automatic detection of corn leaf azimuth angles in field environments based on top-view images captured by drones (He et al., 2024)Yuhao Qing et al. constructed a YOLO network based on an improved RepVGG and combined it with CSL technology, transforming the angle regression problem into a classification task, thereby effectively improving the detection accuracy of objects at arbitrary angles in remote sensing images (Qing et al., 2021)These studies indicate that combining YOLO models with angle classification mechanisms can enhance the detection accuracy of crop spatial orientation features, providing technical references and methodological foundations for precise measurement of plant spatial posture. Therefore, considering the limitations of existing segmentation-based models in spatial posture estimation, this paper does not use semantic segmentation frameworks such as Mask R-CNN and U-Net to measure the tilt angle of sunflower flower heads. Instead, it proposes a fusion method that combines a lightweight model based on YOLO with geometric analysis to better meet the needs of automated measurement in field environments.

The flower head tilt angle is a critical spatial phenotypic parameter influencing sunflower yield, oil accumulation, and mechanical harvesting efficiency. However, existing measurement methods generally suffer from limited accuracy, low efficiency, and poor environmental adaptability, failing to meet the demands of smart breeding and precision agriculture for high-quality phenotypic data. Therefore, there is an urgent need for a non-contact measurement method that balances accuracy, efficiency, and deployment flexibility to provide foundational data support for flower head angle estimation and precision agricultural operations. To address this, this study proposes a novel sunflower flower head tilt angle measurement method that integrates deep learning, computer vision, and geometric analysis. This method uses non-contact image data input, combining a deep learning model with geometric analysis to address the issues of plant damage, high costs, and low efficiency associated with flower head tilt angle measurement. Specifically, the mask map output by the deep learning model is used to extract the contour of the sunflower head surface and the contour of the stem centerline, serving as the foundation for subsequent geometric analysis. In the geometric analysis phase, the major axis direction of the flower head is first obtained by elliptical fitting of the flower head contour, followed by curve fitting of the stem contour to determine the tangent direction of the stem, thereby estimating the tilt angle. Compared to the baseline deep learning model, this method improves measurement accuracy by 3.7% without increasing equipment costs, thereby enhancing measurement performance. This study not only provides technical support for sunflower cultivation management but also offers potential application value for the development of agricultural automation equipment.

In summary, to achieve the goal of precise measurement of crop phenotypes and digital management of agriculture, it is urgent to develop a practical and scalable solution for measuring the tilt angle of sunflower flower heads. This study addresses this core need by establishing a technical approach focused on non-contact acquisition of tilt angle data. The aim is to ensure field applicability and measurement accuracy while reducing system costs and human interference, thereby achieving efficient and stable data collection. The research design is exploratory in terms of both theoretical framework and method implementation. Its overall goal is to establish a set of methods for intelligent estimation of flower head posture in natural environments and promote the application of related phenotypic parameters in precision agriculture, operation optimization, and equipment design. Specifically, the contributions and sub-objectives of this study are reflected in three aspects: First, in terms of methodological innovation, we propose a measurement process that integrates image recognition and geometric modeling to solve the technical bottlenecks of traditional methods in accurately and quickly accurate representation of spatial posture; second, in terms of performance verification, the stability and accuracy of the method in agricultural environments are evaluated through systematic experiments, and comparisons with existing models and manual methods are conducted to demonstrate its practical value; third, in terms of application expansion, the adaptability of the method in crop phenotyping analysis, intelligent cultivation management, and mechanized harvesting is explored to enhance its operability and scalability in real agricultural scenarios.

In the remaining sections, Section 2 introduces image acquisition, depth models, and geometric analysis methods; Section 3 presents experimental results and analysis; Section 4 discusses limitations and future prospects; and Section 5 summarizes the entire paper.

## 2 Methods

**Figure 1:**
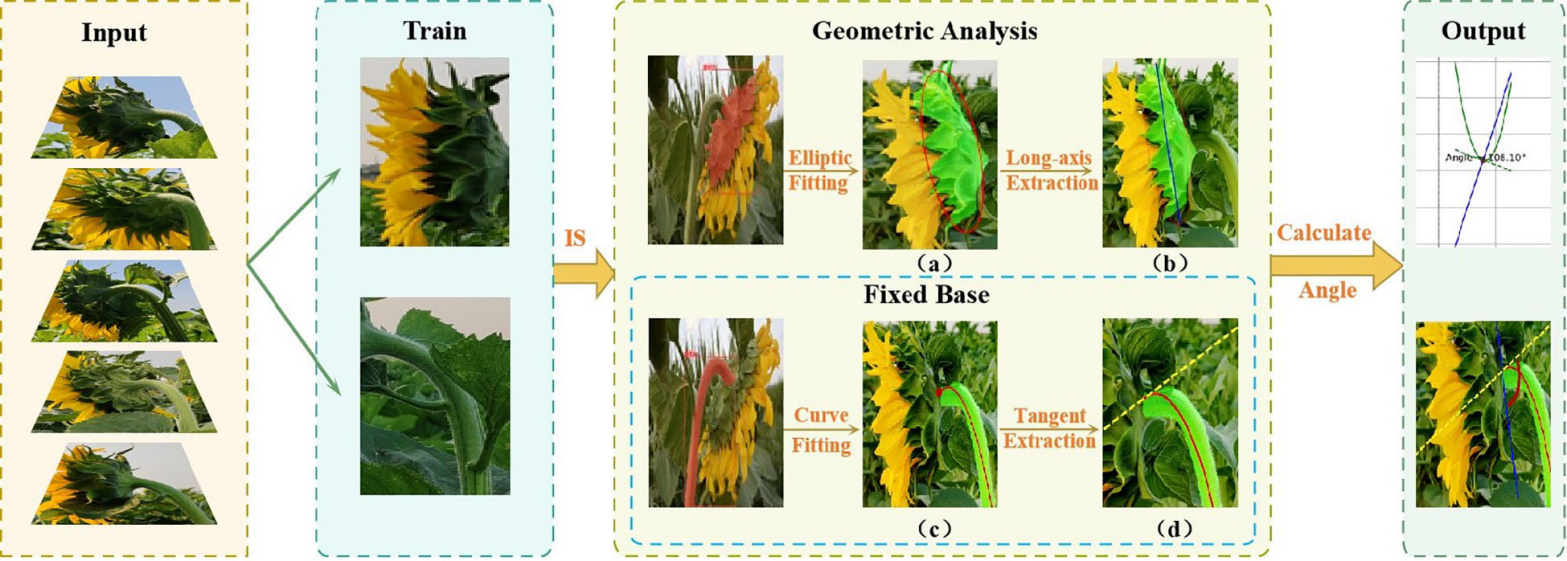
Geometrical analysis process of tilt angle of the disk. **(a)** Elliptical fitting of the sunflower head; **(b)** Extraction of the long axis of the flower head; **(c)** Fitting curve of the stem curvature trend; **(d)** Extraction of the tangent at the intersection point.

This paper proposes a non-contact measurement method that combines deep learning, geometric fitting, and high-precision measurement of the tilt angle of sunflower flower heads. The specific process of this method is shown in **Figure 1**. First, a dataset is constructed, and an improved instance segmentation model is used to accurately extract the sunflower flower head and stem regions. By training a deep learning model, precise identification, segmentation, and classification are achieved, providing reliable data support for subsequent geometric fitting. Subsequently, based on the mask map output by the model, the contour of the sunflower head surface and the centerline contour of the stem are extracted. Ellipse fitting technology is used to determine the reference axis of the head surface. Additionally, curve fitting methods are employed to analyze the bending trend of the stem. Finally, by calculating the angle between the flower head reference axis and the stem tangent, the precise measurement value of the sunflower flower head tilt angle is obtained. The specific implementation steps will be detailed in subsequent sections.

### 2.1 Image acquisition and data preprocessing

The image data used in this study were captured using a Canon EOS 70D digital single-lens reflex camera (equipped with an EF-S 18-135mm f/3.5-5.6 ISSTM lens, resolution of 5472 × 3648 pixels, manual mode, ISO 200, shutter speed of 1/125 second, and aperture of f7.8) at the experimental base of Shanxi Agricultural University. This resolution represents the camera’s original output size, ensuring that all image details are fully preserved. This is of significant importance for subsequent image processing or analysis tasks involving texture, edge, and structural information extraction (Pouliot et al., 2002).All images were captured under natural daylight conditions during daytime, covering different time slots in the morning and afternoon. Although the lighting intensity was not precisely quantified, the natural distribution of shooting times objectively covered a wide range of random lighting conditions, from soft light to strong sunlight, providing a rich sample base for the model to learn features under different lighting conditions. It is important to note that the image acquisition did not include nighttime scenes, and no artificial lighting equipment was used. Therefore, this study focuses on image processing under natural daylight conditions. A total of 1,163 images were obtained, and after quality screening and annotation completeness checks, 1,096 images were retained for model training and testing. The remaining 67 images were not used due to image blur or missing targets. To enhance the objectivity of tilt angle measurements. The samples cover flower head data from different growth angles, spatial directions, and shooting angles, thereby enhancing the diversity of the angle recognition task. All images were sourced from mature sunflowers, excluding early growth stages or periods when flower heads had not yet formed. This is because the primary focus of this study is on detection and recognition applications prior to fruit harvest.

### 2.2 Image enhancement strategies

**Figure 2:**
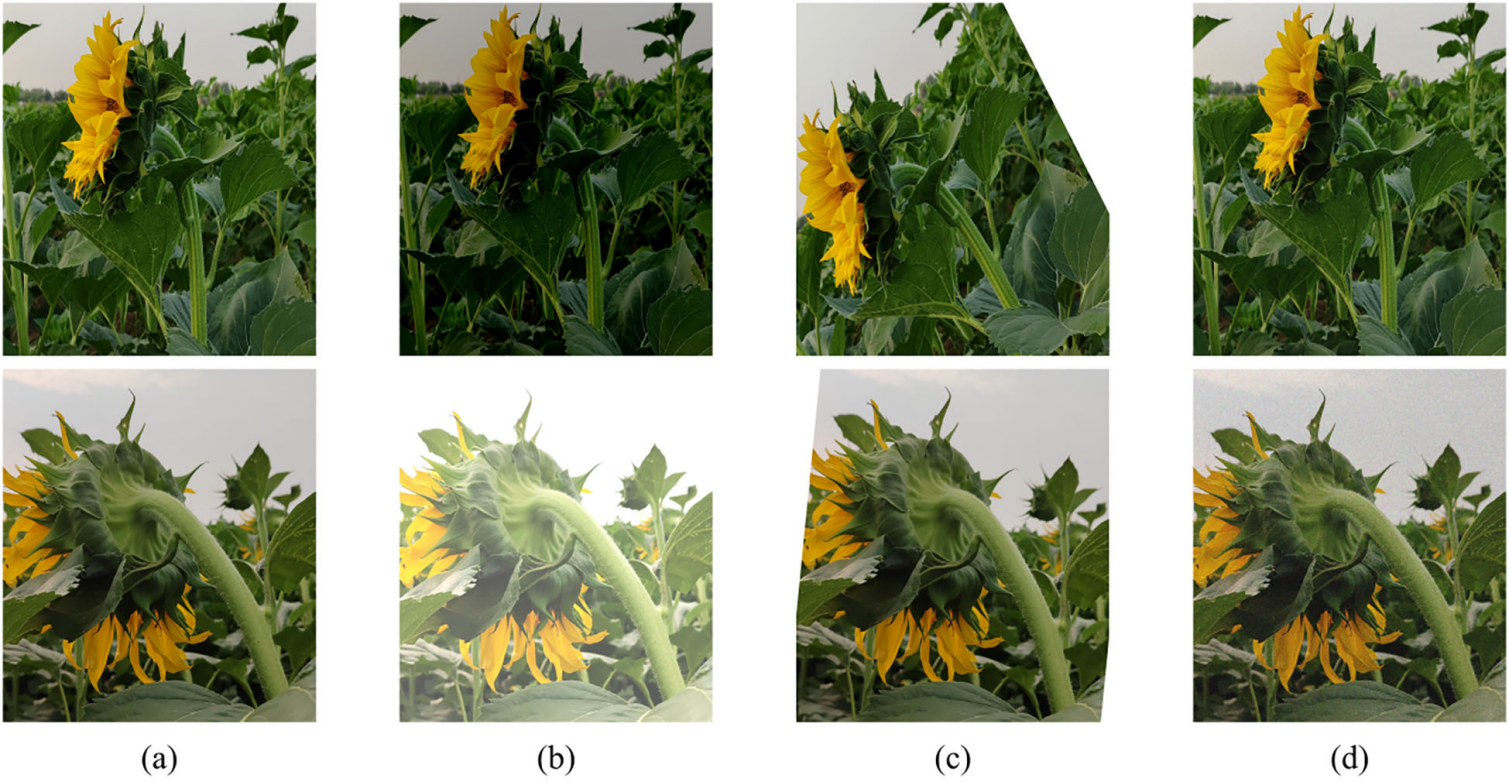
Image enhancement examples. **(a)** Original image; **(b)** Image with different brightness adjustments; **(c)** Image after rotation; **(d)** Image with added Gaussian noise.

To further enhance the model’s robustness in scenarios with varying natural lighting conditions, image enhancement strategies were introduced during the training phase. Due to the complex and variable nature of field environments, images collected under a single environmental condition may not fully reflect the diverse scenarios encountered in practical applications, potentially leading to overfitting of the model to specific image patterns and thereby reducing its generalization ability and robustness across different scenarios. To improve the accuracy and stability of the segmentation model in extracting sunflower flower head and stem contours, this study enhanced the training images using the Albumentations library, employing the following three random enhancement strategies: (1) randomly adjusting brightness within a range of -50% to +50%; (2) randomly rotating the image, with angles varying from -90° to +90°; (3) randomly adding Gaussian noise, with noise levels ranging from 0% to 20%. Images unsuitable for training were excluded, resulting in an expanded training dataset of 2,000 images. The enhanced images retained the original annotation information to ensure consistency with the original data. Related examples are shown in **Figure 2**, where (a) is the original image, (b) shows different brightness treatments, (c) shows image rotation, and (d) shows the addition of Gaussian noise.

### 2.3 Data annotation and set partitioning

Image annotation was performed using Labelme (v4.5.13), with manual polygon-based annotation of the sunflower flower head boundaries, and the results were uniformly converted to YOLO format.To meet the requirements of this study for high-precision annotation of complex edge targets (such as sunflower heads), Labelme was selected as the image annotation tool due to its intuitive operation, simple interface, and suitability for polygon annotation, which facilitates rapid and precise processing of single-category data. In terms of annotation format, the YOLO format was chosen for its real-time and simplicity advantages. Although VOC and COCO provide richer semantic information and more comprehensive structural organization compared to YOLO, their annotation file structures are complex (e.g., XML or JSON), resulting in higher production and parsing costs. Given that this project only involves single-category object recognition and emphasizes lightweight deployment and rapid annotation, a simpler format is more appropriate. Therefore, Labelme combined with the YOLO format achieves a good balance between accuracy and efficiency, better aligning with the practical application objectives of this study. All images are uniformly resized to 640×640 pixels to ensure that target information is visually intact, facilitating efficient model learning. The image dataset was initially randomly divided into training, validation, and test sets in an 8:1:1 ratio, corresponding to 784, 92, and 92 images, respectively. To enhance the representativeness and statistical stability of the samples in the testing phase, the remaining 128 unused images were added to the test set, ultimately expanding the test set to 220 images. Therefore, the final data division ratio is: training set accounts for 67.38%, validation set accounts for 7.91%, and test set accounts for 18.91%. All test images have not participated in any training or validation process to strictly ensure the independence and generalization ability of the model performance evaluation.

### 2.4 Model training settings

Neural network training uses YOLO11 as the base object detection model, primarily due to its excellent balance between accuracy, speed, and deployment performance. YOLO11 is the latest improved version of the YOLO series, incorporating multiple architectural optimizations and training strategies while maintaining a lightweight structure. It excels in detecting small objects and in complex backgrounds. This study was implemented using the PyTorch deep learning framework. The hardware used in this experiment is a 13th Gen Intel(R) Core(TM) i7-13700KF 3.40 GHz processor, 64GB RAM, and an RTX4080 GPU running on the Windows 11 operating system. The software environment includes Python 3.10, PyTorch 1.13, and CUDA 11.7. Various ablation experiments on the model modules were conducted in this environment. During training, the batch size was set to 32, the number of training epochs was set to 200, the SGD optimizer was used, the initial learning rate was set to 0.01, and a cosine annealing strategy was employed for learning rate decay to enhance convergence speed and stability. The loss function selected was Complete IoU (CIoU), which improved the model’s ability to fit and accurately capture target boundaries.

This paper uses PyCharm as the integrated development environment and the Ultralytics framework for model training and inference. OpenCV is used to extract contour regions from segmentation masks, NumPy is used to perform polynomial fitting to extract stem growth trends, and elliptical fitting is combined to extract the long axis direction of the flower head. Based on this, NumPy and SciPy are used to calculate the tilt angle between the flower disk and the stem, and the analysis results are finally visualized using Matplotlib.

### 2.5 Tilt angle data collection and verification method

**Figure 3:**
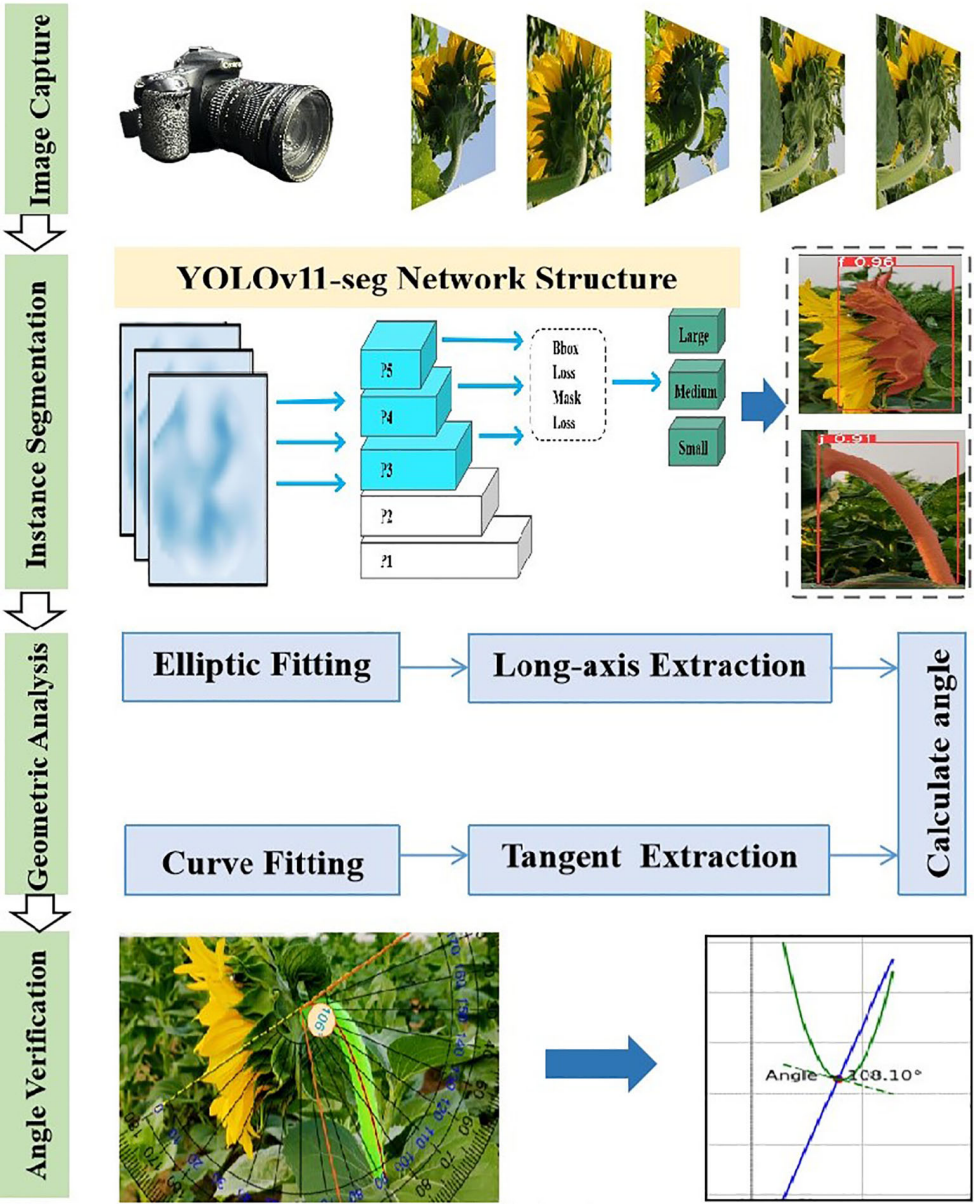
The entire experimental process includes image acquisition, instance segmentation, ellipse fitting, major axis extraction, stem curve fitting, tangent extraction, and inclination angle calculation and verification.

The data acquisition process for tilt angle measurement is depicted in the ‘Fixed Base’ section, as shown in **Figure 1**.First, a curve is fitted to the stem region, and then the tangent direction of the stem fitting curve at the intersection with the flower head is determined, which serves as the measurement reference. The red curve is obtained by fitting a cubic polynomial and is used to characterize the bending trend of the stem. The yellow dashed line indicates the tangent direction calculated at the intersection point. Finally, align the online protractor with the fixed reference point and collect the tilt angle data of the sunflower flower head, as shown in the”Angle Verification”section of **Figure 3**. The protractor displays the measured tilt angle of the flower head (106°). Measure the tilt angle twice using the online protractor, and take the average of the two measurements as the final measurement result. **Table 1** shows the 10 randomly selected datasets from the complete test set, with the following field definitions: “Plant ID” is the unique identifier of the plant from which the data was collected; “Measured Angle (Trial 1)” is the first measured stem tilt angle (unit: °); “Measured Angle (Trial 2)” is the stem tilt angle measured during the repeat measurement (unit:°); In this study, the”Mean Measured Angle (°)” obtained from two independent measurements conducted under consistent conditions using the online protractor tool was used as the ground truth. This averaging method reduces the influence of manual measurement noise (Hoffman and Frankel, 2018), with an average measurement error of 3.83°and a MAE of 1.99°. Additionally, the tangent direction at the intersection of the stem-fitted curve and the flower head is determined by the algorithm, objectively and consistently establishing a reference baseline to reduce inter-operator variability. This enhances the robustness and reproducibility of the measurement process, providing a reference for using the average measurement angle as the baseline in subsequent model evaluations. Therefore, this angle value can serve as the initial reference true value in this study. The “Predicted Angle (°)” refers to the angle value output by the model.

**Table 1:** Sample of measured and predicted data for inclination angle of some sunflower disks.

Plant ID	Measured angle (Trial 1)	Measured angle (Trial 2)	Mean measured angle (°)	Predicted angle (°)
1	100	106	103	102.7
2	88	92	90	91.2
3	92	91	91.5	95.2
4	76	75	75.5	72.7
5	93	100	96.5	101.5
6	77	71	74	74.6
7	79	73	76	77.4
8	91	99	95	100.3
9	79	87	83	77
10	96	105	100.5	101

**Figure 4:**
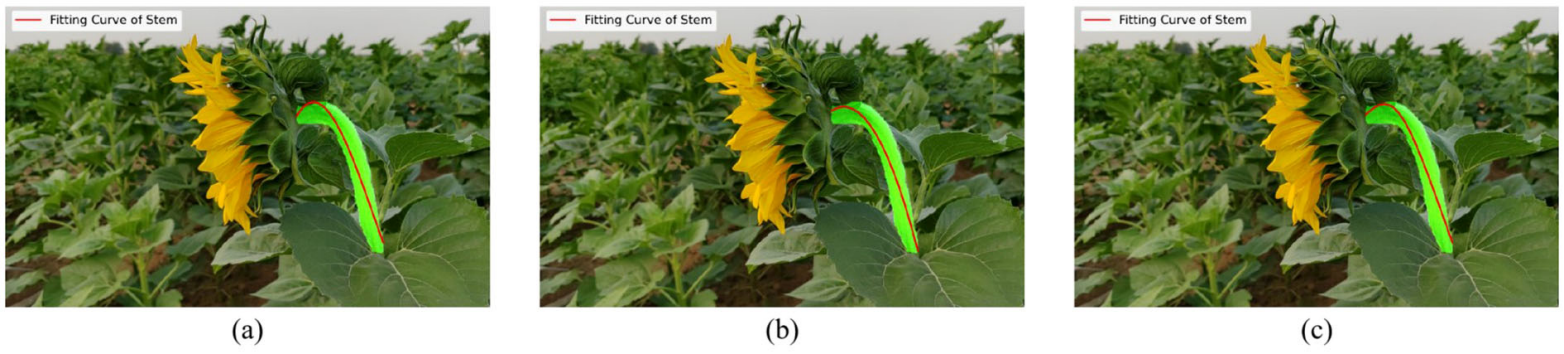
Stem curve fitting with polynomial models of different orders. **(a)** Second-order polynomial fitting curve; **(b)** Third-order polynomial fitting curve; **(c)** Fourth-order polynomial fitting curve.

To explain the parameter settings of the fitting algorithm, this paper uses the least squares method to fit the stem centerline data and compares the fitting effects of polynomials of different orders. Second-order, third-order, and fourth-order polynomials were used for fitting, with the results shown in **Figure 4**. **Figures 4a–c** correspond to the second-order, third-order, and fourth-order fitting models, respectively. Based on the stem centerline data extracted from the current image, the overall shape of the stem changes smoothly, and the curvature changes are relatively simple. A third-order polynomial is sufficient to accurately describe its main bending trend. The fitting results are shown in **Figure 4**. The second-order polynomial fitting results (**Figure 4a**) cannot accurately fit the bending trend at the top of the stem, exhibiting obvious fitting deficiencies. Although the fourth-order polynomial (**Figure 4c**) increases the degree of freedom, its fitting results are similar to those of the third-order polynomial, falling under the category of “increased model complexity with limited benefits” (Ståhl et al., 2021).Additionally, higher-order models, while improving fitting complexity, are prone to overfitting risks (Broersen, 2002), especially when fitting samples from different plants or under different growth conditions, where local regions may exhibit unnecessary oscillations. Furthermore, the third-order polynomial fitting results (**Figure 4b**) have fewer model parameters, resulting in higher computational efficiency and better suitability for subsequent data processing and practical applications (Tong et al., 2021). Therefore, under similar fitting performance, lower-order models were prioritized. Based on the above analysis, all stem curve fittings in this study were performed using third-order polynomial models.

### 2.6 Geometric angle calculation

#### 2.6.1 Curve fitting of stalks

In order to more accurately describe the bending tendency of the stalks and to improve the ability to portray complex curve patterns, this paper adopts the third degree polynomial to fit the curves and optimizes the coefficients by the least squares method. Compared with the traditional simple curve fitting method, this method has higher accuracy and adaptability in the ability to capture nonlinear variations and the portrayal of local geometric features. Specifically, in this study, a third degree polynomial is first fitted to the contour point coordinates and the best coefficients are solved using the least squares method to optimize the curve fitting. The derivatives of the fitted curves are then utilized to reflect the local changes of the stalks, to reduce the visual observation errors and to improve the accuracy of the geometric attitude analysis (Palme, 2006). Based on this, the mathematical formulation of the fitted curve is as follows:


(1) $$y_{s}=a_{0}+a_{1}x+a_{2}x^{2}+a_{3}x^{3}$$

Where, in Equation 1 is the vertical coordinate of the stem curve; is the horizontal coordinate; and is the fitting coefficient, which is optimized by minimizing the sum of squares of the errors between the data points and the fitted curve:


(2) $$Min\overset{n}{\underset{i=1}{\sum }}{\left(y_{s_{i}}-\left(a_{0}+a_{1}x_{i}+a_{2}x_{i}^{2}+a_{3}x_{i}^{3}\right)\right)}^{2}$$

$\left(x_{i},y_{si}\right)\;$ in Equation 2 is the collected stalk curve data points; n  is the number of data points. The red fitted curve demonstrates the bending trend of the stem culm as shown in (c) in **Figure 1**.

#### 2.6.2 Disk ellipse fitting and long axis extraction

This study did not use traditional preprocessing steps (such as filtering and smoothing) to handle boundary noise before elliptical fitting. Instead, we adopted a strategy that combines image enhancement and automated fitting to directly model the target area as an ellipse. During the training phase, we designed diverse data augmentation operations to enhance the model’s robustness against boundary noise and image variations. Specifically, these included randomly adjusting image brightness, randomly rotating images, and adding Gaussian noise with random intensity.

In geometric analysis of flower discs, it is a challenge to accurately extract boundary features and reduce noise interference. Compared with traditional methods, ellipse fitting through least squares can reflect the main structural features of the disk more stably (Jin, 2020). And it shows more adaptability in dealing with irregular contours. Moreover, the traditional method may lead to a large error in the extraction of the long axis when the boundary is irregular, while the present method calculates the long axis direction through the rotation angle, which avoids the error caused by the assumption on the fixed direction and ensures the accuracy of the long axis direction. Further, the accurate extraction of the direction vector is ensured by converting the rotation angle to radians and combining it with trigonometric functions to calculate the long axis endpoints. Ellipse fitting is used to describe the geometrical properties of the disk boundary as a mathematical approximation model of the boundary, which is particularly suitable for regularly shaped disks. In the case of a noisy boundary, the method can still stably extract the main structural features of the disk. In addition, for the case of large boundary fluctuations, the fitting model can be optimized or the boundary processing method can be adjusted to improve the measurement accuracy. Therefore, the general quadratic representation of the ellipse:


(3) $$Ax^{2}+Bxy+Cy^{2}+Dx+Ey+F=0$$

The coefficients of the elliptic equation are in Equation 3 A, B, C, D, E, F. These parameters determine the shape, position, and rotation angle of the ellipse. The final fit uses Equation 4 to minimize the error at the sampling boundary:


(4) $$Min\overset{n}{\underset{i=1}{\sum }}{\left(Ax_{i}^{2}+Bx_{i}y_{i}+Cy_{i}^{2}+Dx_{i}+Ey_{i}+F\right)}^{2}$$

To ensure that the fit results in an ellipse, the following geometric constraints need to be satisfied as Equation 5:


(5) $$4AC-B^{2}>0$$

After determining that the fitted curve is elliptic, the general quadratic equation can be converted to the standard elliptic equation by then optimizing the parameters obtained by the least squares method. The standard equation of an ellipse:


(6) $$\frac{{\left(x-h\right)}^{2}}{a^{2}}+\frac{{\left(y-k\right)}^{2}}{b^{2}}=1$$

After normalizing the elliptic equation as shown in Equation 6, the following key geometric parameters can be extracted: (h,k) denotes the center of the ellipse, which represents the geometric center of the ellipse and approximately corresponds to the center position of the disk; a is the length of the long axis, and b is the length of the short axis, which represent the maximum and minimum diameters of the ellipse, respectively.

The angle of rotation is calculated according to Equation 7 to indicate the degree of inclination of the long axis of the ellipse with respect to the horizontal axis, and θ indicates the main inclination direction of the disk. According to the coordinate axis rotation derivation process described in OpenStax Pre-Calculus Section 10.4, the rotation angle of the ellipse can be obtained using the following formula:


(7) $$cot\left(2\theta \right)=\frac{A-C}{B}$$

This can be further derived into a commonly used form as shown in Equation 8:


(8) $$\theta =\frac{1}{2}tan^{-1}\left(\frac{B}{A-C}\right)$$

As shown in **Figure 1a**, the red ellipse is the fitting result of the disk.

After obtaining the geometrical parameters of the ellipse, the long axis direction can be further extracted to determine the main inclination trend of the disk. The extraction of the long-axis direction is based on the rotation angle and center point of the fitted ellipse, which can accurately portray the geometric attitude of the disk. The long axis direction is determined by the rotation angle θ of the fitted ellipse. To facilitate the calculation, the rotation angle is first converted to the radian system using Equation 9:


(9) $$\theta_{rad}=\theta \times \frac{\pi }{180}$$

$\theta_{rad}\;$ represents the radian value of the angle, and then the direction vector of the long axis is calculated according to Equation 10:


(10) $$\vec{v}=\left(cos\theta_{rad},sin\theta_{rad}\right)$$

This direction vector reflects the main direction of inclination of the disk. Next, the slope $k_{l}$ of the long axis can be calculated using Equation 11:


(11) $$k_{l\;}=tan\left(\theta_{rad}\right)$$

The angle θ between the long axis direction and the horizontal axis is used to describe the spatial attitude of the disk. Then the coordinates of the two endpoints of the long axis are calculated according to the direction and length of the long axis using Equation 12 and 13, respectively:


(12) $$\left(x_{1},y_{1}\right)=\left(x_{0}+\frac{a}{2}cos\theta_{rad},y_{0}+\frac{a}{2}sin\theta_{rad}\right)$$

Long axis endpoint 1:

Long axis endpoint 2:


(13) $$\left(x_{2},y_{2}\right)=\left(x_{0}-\frac{a}{2}cos\theta_{rad},y_{0}-\frac{a}{2}sin\theta_{rad}\right)$$

These two long-axis endpoints start from the center of the ellipse and extend in the long-axis direction to each side for half the length of the long-axis. This calculation process ensures the consistency of the long axis direction with the geometric properties of the ellipse. Finally, by calculating the slope and endpoints of the obtained long axis, the linear equation of the long axis can be obtained Equation 14:


(14) $$y=k_{l\;}x+c_{l\;}$$

This equation represents the mathematical description of the long axis of the disk, as shown in the (b) diagram in **Figure 1**, with the blue line being the direction of the extracted long axis.

#### 2.6.3 Intersection calculation

After fitting the ellipse as the disk boundary, the red intersection point shown in the (c) plot in **Figure 1** is the intersection point between the stem curve and the disk boundary. The location of the intersection point is determined by calculating the intersection of the fitted curve with the ellipse boundary. This demonstrates the spatial relationship between the two and provides the basis for subsequent tangent direction calculations. Confirmation of the location of the intersection point requires solving the expression for the third degree polynomial fit of the stem curve in conjunction with the standard elliptic expression for the disk boundary as shown in Equation 15:


(15) $$\{\begin{array}{c} y=a_{0}+a_{1}x+a_{2}x^{2}+a_{3}x^{3}\\ \frac{{\left(x-h\right)}^{2}}{a^{2}}+\frac{{\left(y-k\right)}^{2}}{b^{2}}=1 \end{array}$$

#### 2.6.4 Tangent direction of the stem curve

All geometric fitting processes involved in this method are automatically executed by the algorithm without any manual intervention. Specifically, the bending trend of the stem is modeled by fitting a cubic polynomial curve using the least squares method. Based on this, the algorithm automatically determines the tangent reference points at the intersection of the elliptical fit boundary of the flower disk and the stem fit curve, and calculates the tangent direction at that position, which serves as the reference for subsequent inclination measurements, without any manual setup or adjustment throughout the process. The slope of the tangent at the intersection point is automatically calculated using the first derivative expression of the fitted curve, accurately reflecting the local geometric trend at that position. The first derivative form of the cubic polynomial is shown in Equation 16.


(16) $$f^{\prime }\left(x\right)=3a_{3}x^{2}+2a_{2}x+a_{1}$$

Use Equation 17 to find the slope of the tangent line at the point of intersection as:


(17) $$k_{s\;}=f^{\prime }\left(x\right)$$

The yellow dashed line indicates the tangent line at the intersection, as shown in the (d) diagram in **Figure 1**.

#### 2.6.5 Tilt angle calculations

The angle of inclination reflects the relative spatial relationship between the tangent line of the stalk and the long axis of the disk, specifically calculated from the slopes of the two straight lines. The formula is:


(18) $$\theta =arctan\left(\left|\frac{k_{l}-k_{s}}{1+k_{l}k_{s}}\right|\right)$$

Where $k_{l}$ in Equation 18 is the slope of the long axis of the ellipse; $k_{s}$ is the slope of the tangent line to the stem curve at the intersection.

### 2.7 Model design and optimization

In the traditional segmentation network architecture used in the early stages of the experiment, the large parameter size and high computational cost made it difficult to effectively capture the complex and variable angles of sunflower heads, thereby limiting the network’s adaptability in instance segmentation tasks. These issues made it challenging to achieve model lightweighting while maintaining accuracy and robustness. To address this, this paper proposes two improved modules based on the YOLO11-seg structure: CKB (Cross Stage Partial Kernel with Reparameterized Vision Transformer Block) and CKBM (Cross Stage Partial Kernel Reparameterized Vision Transformer Block with Efficient Multi−Scale Attention). By combining RepViTBlock with the EMA attention mechanism, these modules reduce the number of parameters and computational complexity while effectively enhancing the model’s ability to express instance-level features and its generalization performance.

Specifically, the backbone network of the YOLO series algorithms typically uses Bottleneck modules for local feature extraction. Although this structure performs well in simple scenarios, its modeling capabilities are limited when dealing with the complex tilt angles of sunflower heads. This limitation impacts the model’s stability and accuracy in critical tasks such as boundary identification and instance discrimination. To address this issue, the CKB module designed in this paper introduces the RepViTBlock, which possesses global modeling capabilities, while integrating Deep Separable Convolution (DW) and Channel Attention Mechanism (SE), effectively enhancing the model’s ability to model multi-scale and fine-grained features. The module achieves channel expansion and compression through two 1×1 convolutions and introduces residual connections to maintain gradient propagation stability and information integrity, thereby enhancing the network’s performance in complex crop instance segmentation scenarios.

To visually demonstrate the optimization process of the network structure, **Figures 4a** and **1b** show the comparison between the standard structure and the optimized structure, respectively. The original structure primarily consists of convolutional layers (Conv) and multi-layer C3K2 modules, while in the optimized structure, all replaced modules are highlighted with dashed borders for clear identification. In the backbone section, the original C3K2 is replaced by CKB and CKBM to enhance the context modeling capability of feature extraction. In the neck section, the original multi-scale fusion strategy is retained, and the original C3K2 module is also replaced by CKB to improve the interaction efficiency of semantic and structural information, thereby enhancing the modeling capability of the output branch for target boundaries and instance differences. The overall structure maintains the original network topology while enhancing instance perception capabilities through module replacement, effectively improving the model’s segmentation accuracy and structural expression capabilities in sunflower instance segmentation tasks.

**Figure 5:**
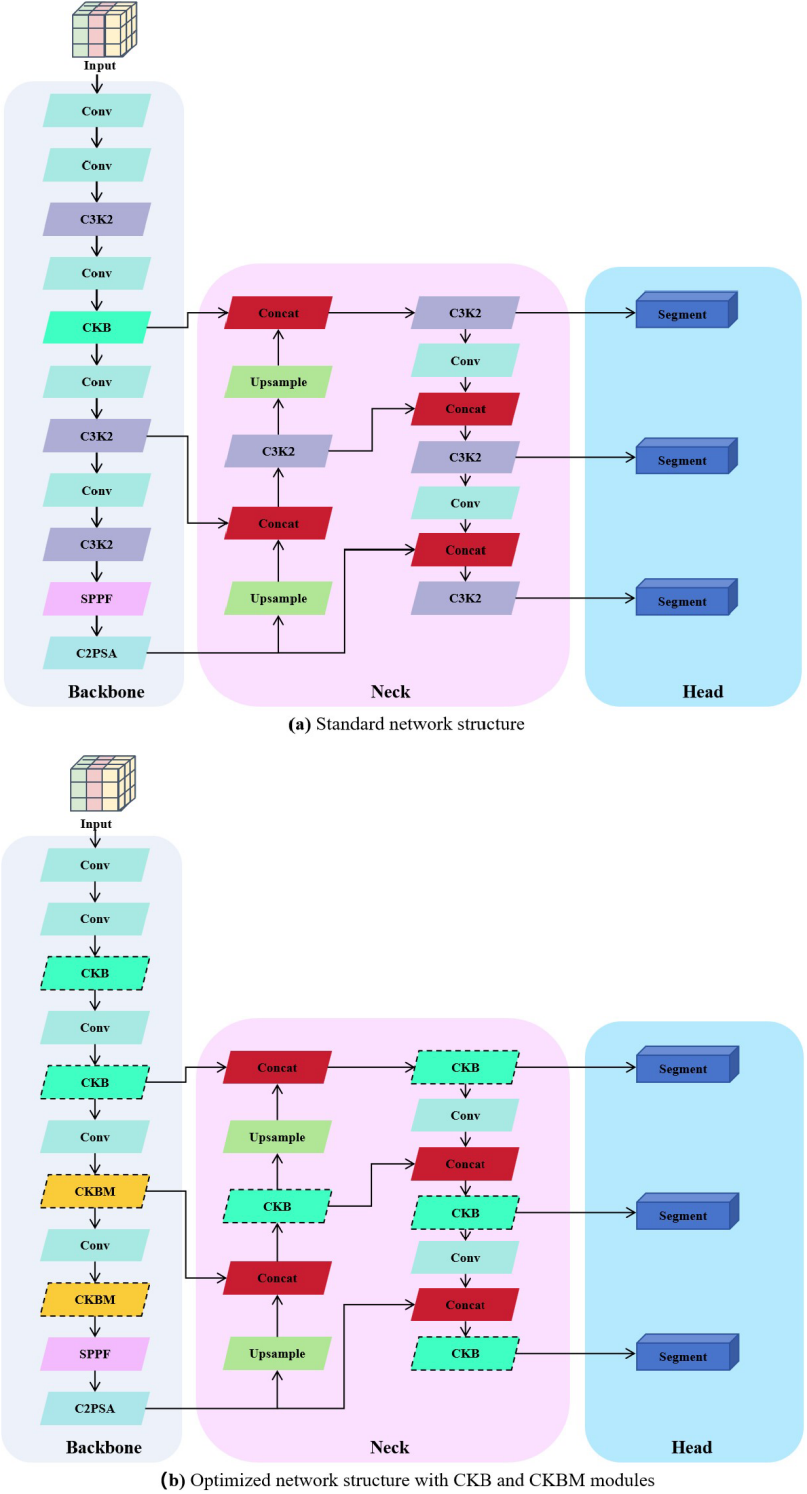
Comparison between standard and optimized structures incorporating CKB/CKBM modules. **(a)** Standard network structure; **(b)** Optimized network structure with CKB and CKBM modules.

In the YOLO series of algorithms, the backbone network mainly uses the Bottleneck module to extract local features. This module can extract certain features in simple scenes, but has limited performance in complex growth patterns. This limitation affects the accuracy and stability of the model in the task of disk tilt angle measurement. To address this problem, this study proposes the CKB module based on the RepVit module. This module combines a depth-separable convolutional DW and SE attention mechanism. By introducing the RepViTBlock with global information processing capability to replace the Bottleneck module, the network’s ability to extract key features of sunflowers is enhanced. The design of the module uses two 1×1 convolutions to realize channel expansion and compression, while residual connections are introduced to guarantee the information transfer efficiency and stability of model training. This design enhances the model’s ability to segment the complex growth patterns of sunflower disks and stalks. The specific network structure is shown in **Figure 5**.

**Figure 6:**
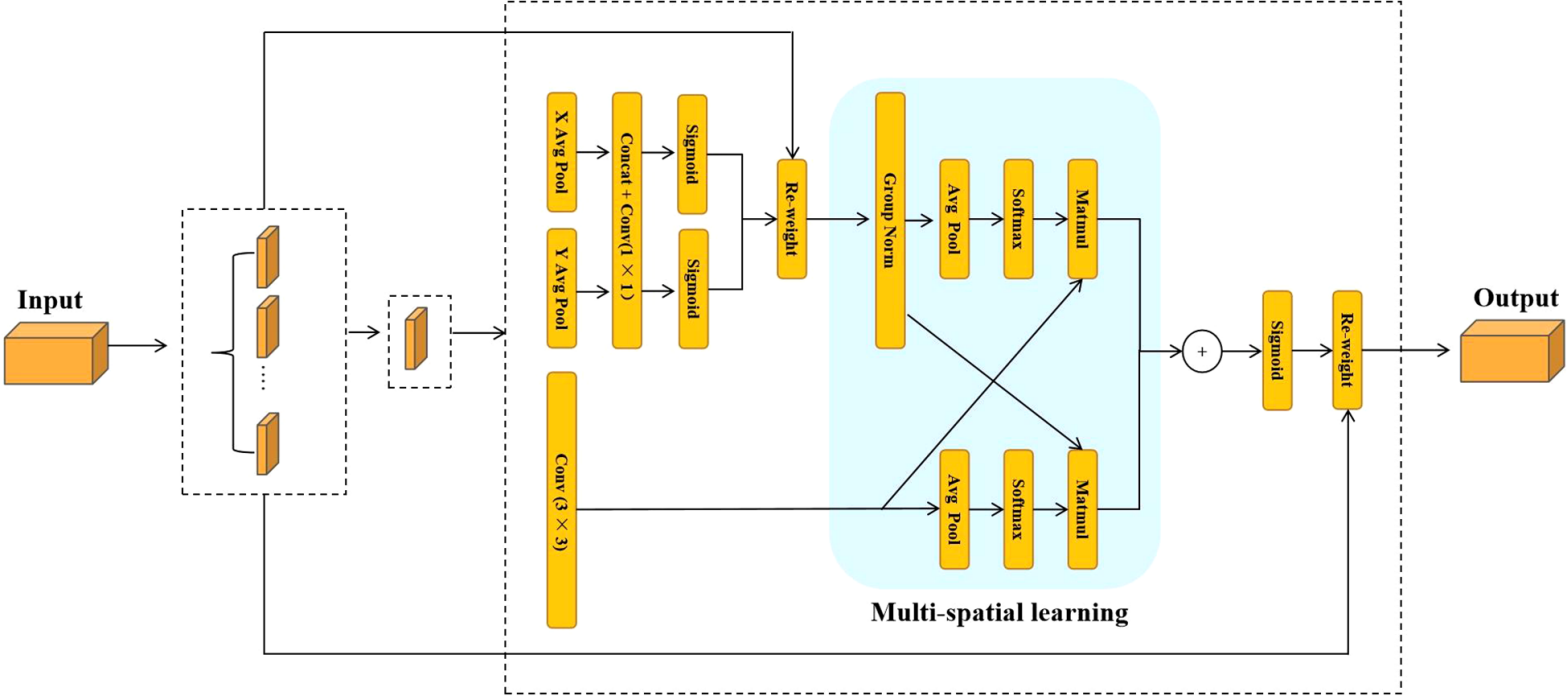
Structure of the EMA attention mechanism.

**Figure 7:**
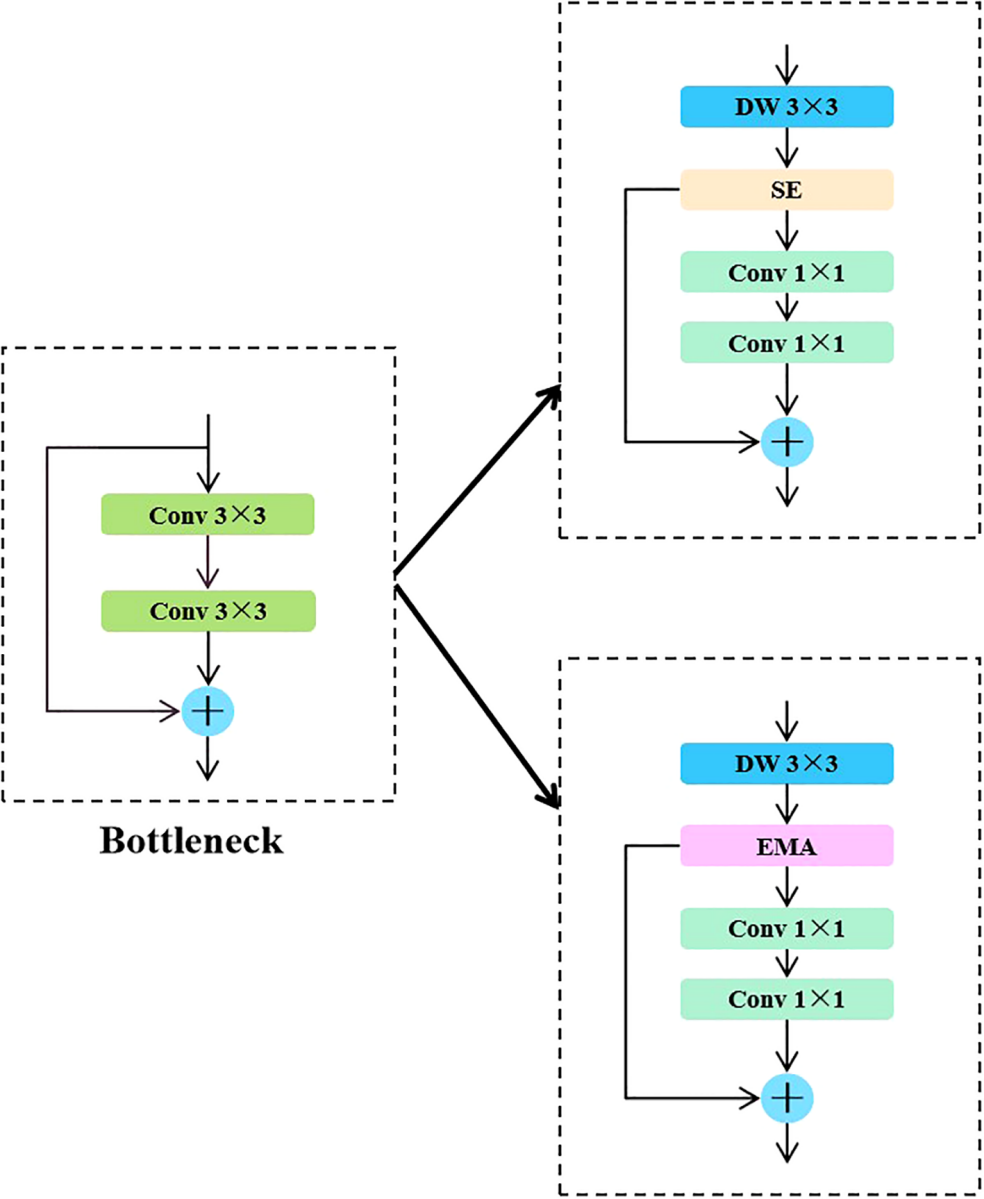
Module replacement flow.

On the basis of CKB, this study further proposes the CKBM module, which strengthens the global context modeling capability of the network by replacing the SE attention mechanism in the RepViTBlock module with the EMA attention mechanism, which is capable of jointly modeling the spatial and channel dimensional features to provide a finer-grained attention allocation for the segmentation region of sunflower. This improvement enhances the accuracy of the boundaries of the sunflower segmentation region, improves the generalization ability of the model, and makes it more suitable for the task of measuring the inclination angle of sunflower discs with diversified growth patterns. The structure of the EMA attention mechanism and the process of module substitution are shown in **Figures 6** and **7**, respectively.

### 2.8 Evaluation indicators

The model performance was assessed by three metrics, RMSE, MAE and R², which measure the overall error, the mean deviation and the goodness of fit, respectively (Ang et al., 2020). These metrics are used to quantitatively analyze the performance of the model in predicting angles on the test set. The results can assess the accuracy of the sunflower disk tilt angle measurement method, as well as provide a reference for subsequent optimization.

#### 2.8.1 Root mean square error

In order to quantify the overall error between the model’s predicted angle and the actual measured angle, this paper uses the root mean square error (RMSE) as an evaluation metric (Bao et al., 2023). RMSE denotes the square root of the squared mean of the error, which assigns a higher weight to larger deviations (Entekhabi et al., 2010). In the task of sunflower disk tilt angle measurement, due to the complexity of growth morphology and significant individual differences, RMSE can accurately quantify the overall error level of the model, which is especially obvious on disks with special growth morphology. Its calculation formula is as follows:


(19) $$RMSE=\sqrt{\frac{1}{n}\overset{n}{\underset{i=1}{\sum }}|{\left(y_{i}-{\hat{y}}_{i}\right)}^{2}}$$

where $y_{i}$ in Equation 19 denotes the actual angle, ${\hat{y}}_{i}\;$ denotes the predicted angle, and n is the total number of samples.

#### 2.8.2 Mean absolute error

In order to assess the average deviation between model predictions and actual measurements, this paper uses the mean absolute error (MAE) as a key indicator. MAE intuitively reflects the average size of the model errors, assigns equal weight to all errors, is not affected by extreme errors, and can measure the stability and consistency of the model (Hodson, 2022). In the measurement of sunflower disk tilt angle, MAE can accurately reflect the prediction performance of the model in complex scenarios due to the large differences in growth patterns and tilt angles of different disks. Its calculation formula is as follows:


(20) $$MAE=\frac{1}{n}\overset{n}{\underset{i=1}{\sum }}|y_{i}-{\hat{y}}_{i}|$$

where $y_{i}$ in Equation 20 represents the actual angle, ${\hat{y}}_{i}\;$ is the predicted angle, and n is the total number of samples.

#### 2.8.3 Coefficient of determination

In order to assess the ability of the model to explain the trend of tilt angle changes, this paper uses the coefficient of determination as an evaluation index. $R^{2}\;$ indicates the degree of fit between the model’s predicted value and the actual value, with the value ranging from 0 to 1. The closer the value is to 1, the stronger the model’s ability to explain the trend of angular changes, and the better the fitting effect is. In sunflower disk tilt angle measurement, the tilt angle differences between different disks are complex and have dynamic changes, $\;R^{2}$ can quantify the model’s ability to capture these trends. Its calculation formula is as follows:


(21) $$R^{2}=1-\frac{\sum_{i=1}^{n}{\left(y_{i}-{\hat{y}}_{i}\right)}^{2}}{\sum_{i=1}^{n}{\left(y_{i}-\bar{y}\right)}^{2}}$$

where $y_{i}$ in Equation 21 denotes the actual angle, ${\hat{y}}_{i}\;$ denotes the predicted angle, ${\bar{y}}_{i}\;$ is the mean value of the actual angle, and n is the total number of samples.

## 3 Results and analysis

### 3.1 Model performance analysis

**Figure 8:**
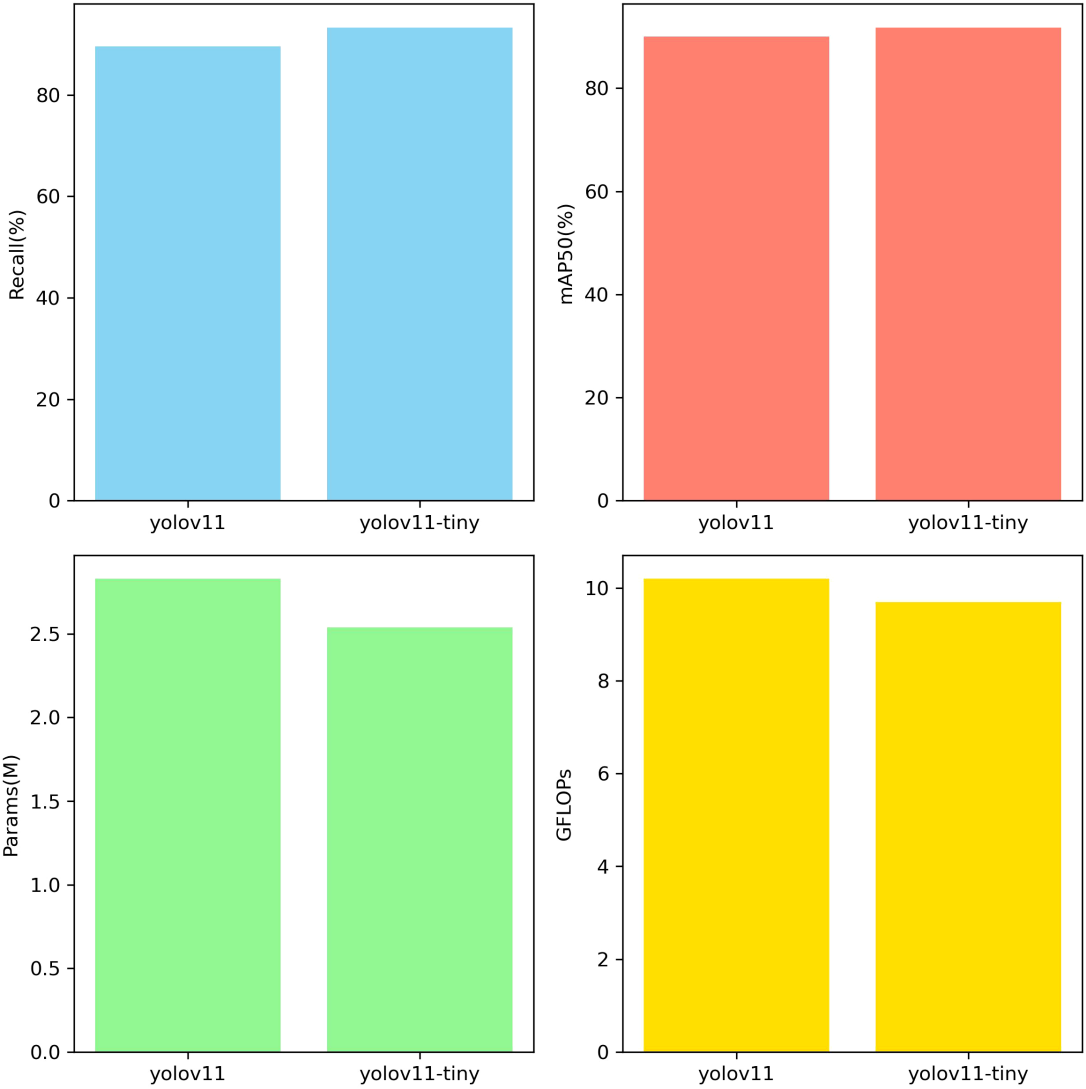
Performance evaluation.

**Figure 9:**
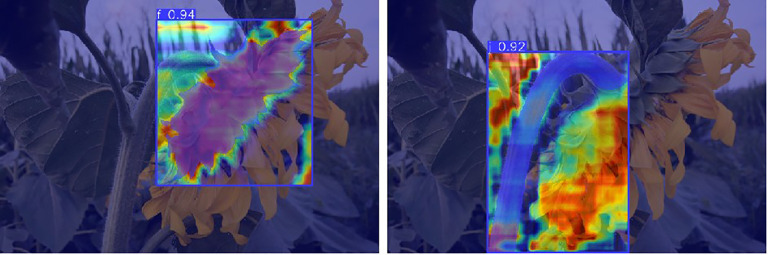
Example segmentation effect of flower disk and stem.

In this study, model performance was evaluated using a test set. The test set data was not used for training and was solely used to validate model performance. Evaluation metrics included recall rate, mAP50, number of parameters, and GFLOPs. **Figure 8** shows the evaluation results, clearly validating the model’s performance and efficiency in the sunflower disk tilt angle measurement task. The evaluation results indicate that the improved YOLO11-seg model outperforms the original YOLO11 model. Recall improved by 3.7%, and mAP50 improved by 1.8%. The number of parameters decreased by 0.29M, and the number of floating-point operations decreased by 0.5GFLOPs (i.e., the model reduces approximately 5×10^8^ floating-point operations per image inference). **Figure 9** shows the instance segmentation results for the disk surface and stem. These improvements indicate that the optimized model reduces the number of parameters and computational complexity.

To more comprehensively evaluate the model’s segmentation capabilities for different target structures and to address the research requirement for identifying different parts of sunflowers, performance assessments were conducted on the flower head and stem under the same model architecture and training strategy. The segmentation performance results are shown in the **Table 2** below. Due to the complex morphology and irregular boundaries of the flower head, dense polygon points were required during annotation to improve shape accuracy; in contrast, the stem has a linear structure with clear boundaries, allowing it to be described using fewer annotation points. The evaluation results show that the Recall and mAP50 values for the flower head are slightly higher than those for the stem, consistent with the differences in structural complexity and annotation accuracy between the two. Overall, the model demonstrates good segmentation performance for both the flower head and stem.

**Table 2:** Segmentation performance metrics for sunflower head and stem.

Part	Recall	mAP50
Sunflower Head	0.949	0.917
Sunflower Stem	0.936	0.904

### 3.2 Model comparison experiment

To validate the effectiveness of the proposed model in sunflower instance segmentation tasks, this paper systematically compares it with mainstream segmentation models. Considering that two-stage instance segmentation frameworks (such as the Mask R-CNN series) although they have high segmentation accuracy, they typically have large model sizes, high computational complexity, and slow inference speeds, making them unsuitable for real-time and computationally constrained agricultural application scenarios (Charisis and Argyropoulos, 2024). Given that the proposed model is designed around a single-stage lightweight architecture, primarily targeting edge devices and general-performance terminals in real-world agricultural scenarios, two-stage detection frameworks were not included in the comparison. Instead, the current mainstream YOLO series of lightweight segmentation models were selected as references for systematic performance comparisons.

The comparison models include YOLOv8n-seg, YOLOv8n-seg-p6, YOLOv9c-seg, YOLO11n-seg, and YOLO11s-seg, all of which are representative single-stage segmentation networks, with strong engineering practicality. The YOLOv8n-seg series optimizes feature extraction capabilities through an improved C2f module (Wu et al., 2024), while YOLOv9c-seg introduces the C2f-DW structure, further optimizing feature extraction efficiency and reducing computational complexity (Alanazi, 2025). The YOLO11 series enhances segmentation accuracy by optimizing the backbone network and Neck structure. To comprehensively evaluate model performance, this paper selected Recall, mAP50, Parameters (number of parameters), and GFLOPs (computational complexity) as evaluation metrics in comparative experiments, respectively measuring the model’s segmentation capability, segmentation accuracy, network scale, and computational efficiency. The test results are shown in the table.

In terms of key performance metrics, the proposed model achieved 0.943 and 0.911 for Recall and mAP50, respectively, demonstrating superior target recognition capability and segmentation accuracy. In terms of model complexity, the proposed model contains only 2.54 million parameters and has a computational load of 9.7 GFLOPs, significantly lower than YOLOv9c-seg (27.63 million parameters, 157.6 GFLOPs) and YOLO11s-seg (10.07 million parameters, 35.3 GFLOPs), and outperforms the YOLOv8n series, fully demonstrating its efficiency and lightweight advantages in resource-constrained environments. In summary, the proposed model significantly reduces computational costs while maintaining high segmentation accuracy, offering better practicality and deployment flexibility. The test results are shown in **Table 3**.

**Table 3:** Model comparison test results.

Model	Recall	mAP50	Parameters(M)	GFLOPs(G)
yolov8n-seg	0.927	0.897	3.26	12.0
yolov8n-seg-p6	0.933	0.907	5.09	12.0
yolov9c-seg	0.864	0.857	27.63	157.6
yolo11n-seg	0.908	0.878	2.83	10.2
yolo11s-seg	0.867	0.892	10.07	35.3
**ours**	**0.943**	**0.911**	**2.54**	**9.7**

The bolded values in this Table indicate the results where our method achieved the best performance across all metrics. Specifically, these values demonstrate that our method achieved 0.943, 0.911, 2.54, and 9.7 in Recall, mAP50, Parameters (M), and GFLOPs (G), respectively.

### 3.3 Ablation experiment

In this study, ablation experiments were conducted on the YOLO11n-seg network, and the performance improvements achieved by introducing the CKB and CKBM modules into this architecture were discussed. To comprehensively evaluate the impact of the improved modules on network performance, this section selected Recall, mAP50, Parameters (number of parameters), and GFLOPs (computational complexity) as evaluation metrics, respectively used to measure the model’s detection capability, segmentation accuracy, model size, and inference efficiency. These metrics reflect the actual effects of the proposed improved modules in enhancing model performance, optimizing computational overhead, and achieving lightweight design.

As shown in **Table 4**, when using the CKB module or CKBM module alone in the backbone, both can improve model performance to some extent, but the overall mAP50 performance is still slightly lower than when using both modules together. This is primarily because the CKBM module focuses on enhancing local contextual features and improving instance discrimination capabilities, while the CKB module reinforces global modeling capabilities and multi-scale, fine-grained feature expression. When using either module alone, there are still issues with insufficient feature expression or inadequate instance boundary modeling, which limit further improvements in overall performance. In the T1 experiment, the original YOLO11n-seg structure was used as the baseline, with the C3K2 module retained in both the backbone and neck components. Due to limitations in modeling capability when handling the complex and variable tilt angles of sunflower heads, the model performed poorly in critical tasks such as boundary identification and instance discrimination. with Recall and mAP50 values of 0.908 and 0.878, respectively, and high computational complexity (10.2 GFLOPs), indicating room for optimization. In the T2 experiment, replacing the CKB module in the Backbone significantly enhanced the model’s global modeling capabilities, enabling more thorough feature extraction under complex tilt angles, resulting in notable improvements in Recall and mAP50. Additionally, due to the efficient structural design of RepViTBlock, the number of parameters and computational complexity were further reduced, demonstrating excellent lightweighting effects. In Experiment T3, only the C3K2 module in the Backbone was replaced with the CKBM module, although mAP50 only improved by 0.014 (from 0.878 to 0.892) compared to the original network, the lighter convolutional design of the CKBM module resulted in reduced parameter count and GFLOPs, improving inference efficiency. In Experiment T4, the backbone combines CKB and CKBM modules, while the neck section uses the CKB module, further enhancing overall feature extraction capabilities. This configuration achieves optimal performance in complex instance boundary modeling and instance discrimination, with Recall and mAP50 improving to 0.943 and 0.911, respectively. The number of parameters and computational complexity remain at optimal levels (2.54M, 9.7 GFLOPs), achieving a good balance between model accuracy and inference efficiency. Overall, the improvements in Recall and mAP50 are primarily attributed to the global modeling capabilities provided by CKB and the enhanced local context expression capabilities of CKBM. The reduction in parameter count and GFLOPs is attributed to the design of depth-separable convolutions and EMA attention mechanisms in the RepViTBlock and CKBM structures. The comparison results of the above four experiments fully demonstrate that the proposed improvement scheme has good practical value and promotion potential in the sunflower instance segmentation task.

**Table 4:** Melting experiment results.

Ablation Setting	yolo11n-seg	CKB	CKBM	Recall	mAP50	Parameters (M)	GFLOPs (G)
T1	**√**			0.908	0.878	2.83	10.2
T2	**√**	**√**		0.922	0.887	2.53	9.7
T3	**√**		**√**	0.915	0.892	2.54	9.8
**T4**	**√**	**√**	**√**	**0.943**	**0.911**	**2.54**	**9.7**

The bolded values in this Table indicate the results where our method achieved the best performance across all metrics. Specifically, these values demonstrate that our method achieved 0.943, 0.911, 2.54, and 9.7 in Recall, mAP50, Parameters (M), and GFLOPs (G), respectively.

This paper uses YOLO11n-seg as the baseline model and compares it with the YOLO11 series models. The model proposed in this paper achieves optimal detection performance with extremely low resource consumption. Its parameter count is only 2.54 million, and its computational complexity is 9.7 G, both of which are lower than the smallest yolo11n-seg (2.83 million and 10.2 G). In terms of performance, the proposed model achieves a Recall of 0.943 and an mAP50 of 0.911, both significantly outperforming larger-scale models such as yolo11n-seg (0.908/0.878) and yolo11l-seg (0.933/0.879). Even when compared to yolo11m/l/x-seg, which has several times more parameters, our model maintains its lead in detection accuracy, demonstrating an excellent balance between performance and efficiency, and showing potential for application in resource-constrained scenarios. Detailed comparison results are shown in **Table 5**.

**Table 5:** Performance comparison between different YOLO11 models and this method in segmentation tasks.

Model	Recall	mAP50	Parameters (M)	GFLOPs (G)
yolo11 n-seg	0.908	0.878	2.83	10.2
yolo11 s-seg	0.867	0.892	10.07	35.3
yolo11 m-seg	0.9	0.903	22.34	123
yolo11 l-seg	0.933	0.879	27.59	141.9
yolo11 x-seg	0.867	0.864	62.0	318.5
**ours**	**0.943**	**0.911**	**2.54**	**9.7**

The bolded values in this Table indicate the results where our method achieved the best performance across all metrics. Specifically, these values demonstrate that our method achieved 0.943, 0.911, 2.54, and 9.7 in Recall, mAP50, Parameters (M), and GFLOPs (G), respectively.

### 3.4 Model learning curve analysis and task adaptability assessment

**Figure 10:**
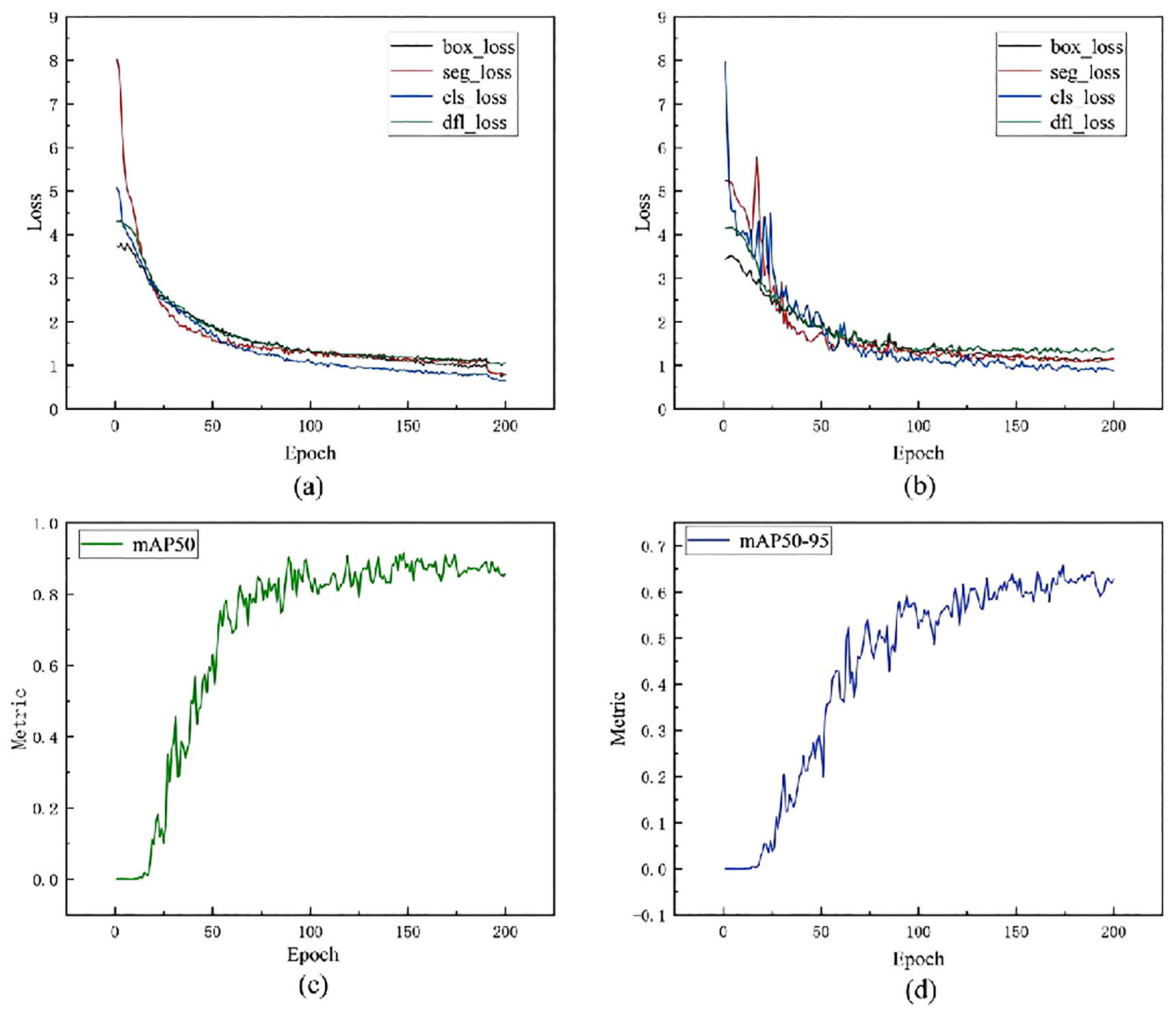
Analysis of learning curves. **(a)** Training loss curve: Shows the changes in box_loss, seg_loss, cls_loss, and dfl_loss as the number of training iterations increases; **(b)** Validation loss curve: Shows the changes in various losses on the validation set, reflecting the model's generalisation ability; **(c)** mAP50 performance curve: Shows the improvement in average precision as training progresses under the condition of IoU=0.5; **(d)** mAP50-95 performance curve; Covers the range of IoU-0.5-0.95, reflecting the model's performance in higher-precision instance segmentation.

The training loss and validation loss curves for the model during the feature learning process, as shown in **Figures 10a, b**, exhibit clear learning characteristics. When combined with the sunflower segmentation task, the following can be summarized: Within the first 20 epochs, all losses (including box_loss, seg_loss, cls_loss, and dfl_loss) show a significant decrease, indicating that the model quickly mastered key features such as target localization, mask boundary segmentation, category classification, and bounding box regression. From 20 to 100 epochs, the curves enter a period of gradual and sustained decline, with training and validation losses highly consistent, indicating that the model has good generalization ability and has not yet exhibited overfitting. From 100 to 200 epochs, the losses tend to converge, implying that the model has approached its performance limit. This training process exhibits the typical characteristics of a “good fit” learning curve, where both training loss and validation loss continue to decrease, and in the later stages of training, they converge and maintain a small error interval, reflecting the model’s good generalization ability and stable training state. Considering the challenges posed by sunflower images, such as complex backgrounds, severe leaf overlap, and diverse flower head angles, this stable convergence further demonstrates that the proposed improved YOLO model can effectively extract the spatial structure and texture boundary information of the target, enabling not only precise target localization but also good segmentation granularity, thereby adapting to flower heads with different growth angles.

**Figure 10c** shows the learning curve of the mAP50 metric during model training. mAP50 represents the average precision under an IoU (Intersection over Union) threshold of 0.50, and is commonly used to evaluate the overall performance of object detection tasks. As shown in the curve, the mAP50 metric of the model increases significantly during the initial training phase, indicating that the model has quickly and effectively learned the key features of the training data. By around 50 epochs, the performance has reached a relatively high level. Subsequently, the mAP50 metric continues to rise slowly until it stabilizes around 150 epochs. This suggests that the model gradually reaches a stable performance state in the subsequent training phase, and further training no longer significantly improves performance.

**Figure 10d** shows the learning curve of the model under the stricter metric mAP50-95, which calculates the average precision from an IoU threshold of 0.50 to 0.95 (with a step size of 0.05), reflecting the model’s higher-precision object detection capability. As shown in the curve, the mAP50–95 metric exhibits a sustained upward trend, indicating that the model progressively improves its ability to precisely locate objects during training. Especially in the early stages (approximately 0 to 50 epochs), the curve shows a significant upward trend, indicating that the model rapidly improves its localization accuracy during this phase. In the middle stage (approximately 50 to 150 epochs), mAP50–95 continues to improve but at a gradually slower pace, reflecting the model’s steadily improving and stabilizing generalization performance. Finally, the curve flattens out after approximately 150 epochs, indicating that the model has reached a relatively stable performance state with limited room for further improvement, suggesting that training is sufficiently adequate. In summary, the learning curves of mAP50 and mAP50–95 collectively demonstrate that the proposed model not only possesses rapid convergence capabilities and a stable learning process but also achieves improvements in both object detection accuracy and boundary localization accuracy. The model reaches performance convergence after 150 epochs, with the number of training iterations set reasonably to avoid overfitting and resource waste, thereby validating the effectiveness and practicality of the model design.

### 3.5 Comprehensive evaluation of angle prediction accuracy

**Figure 11:**
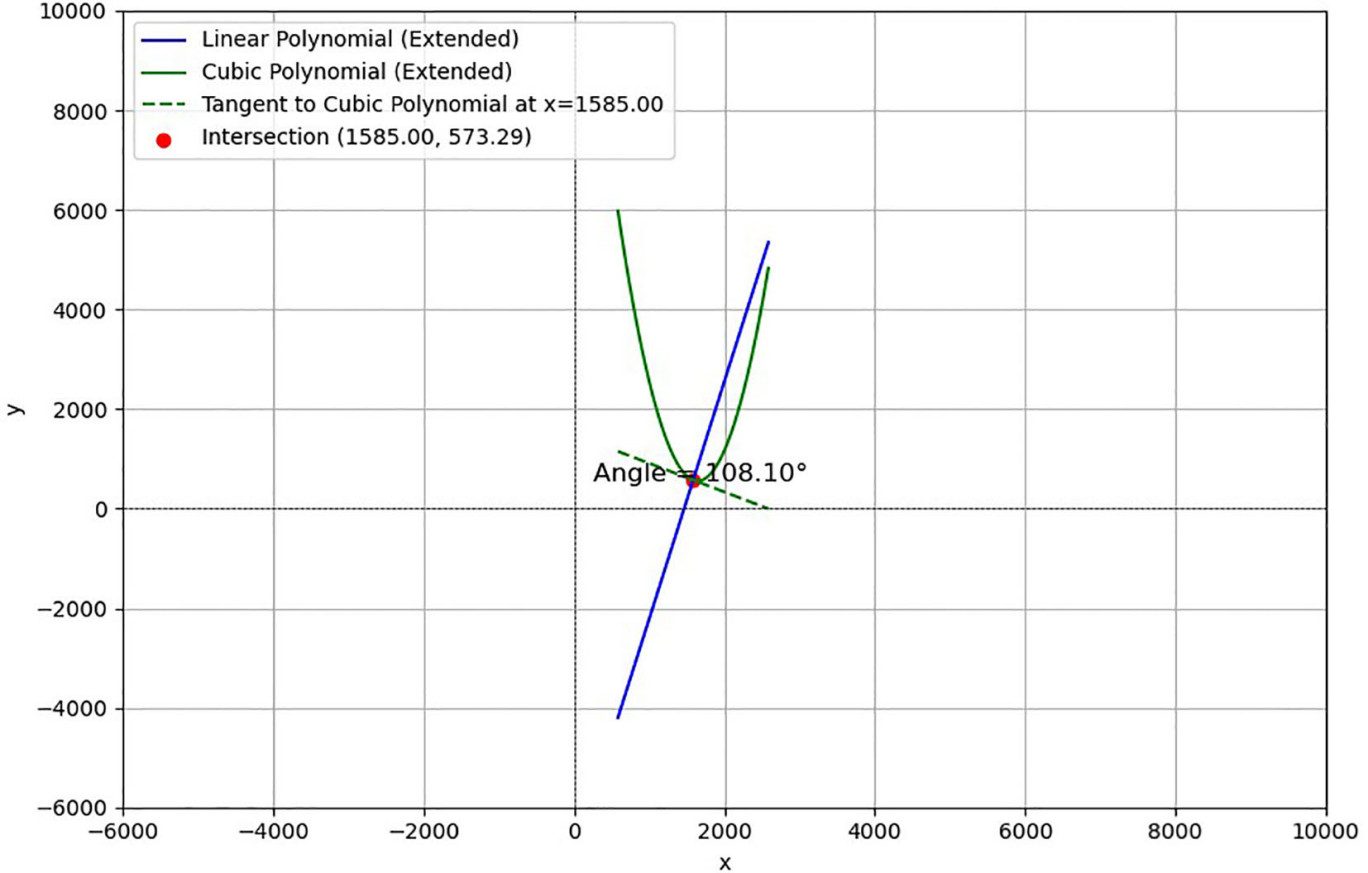
Tilt angle calculation results.

**Figure 12:**
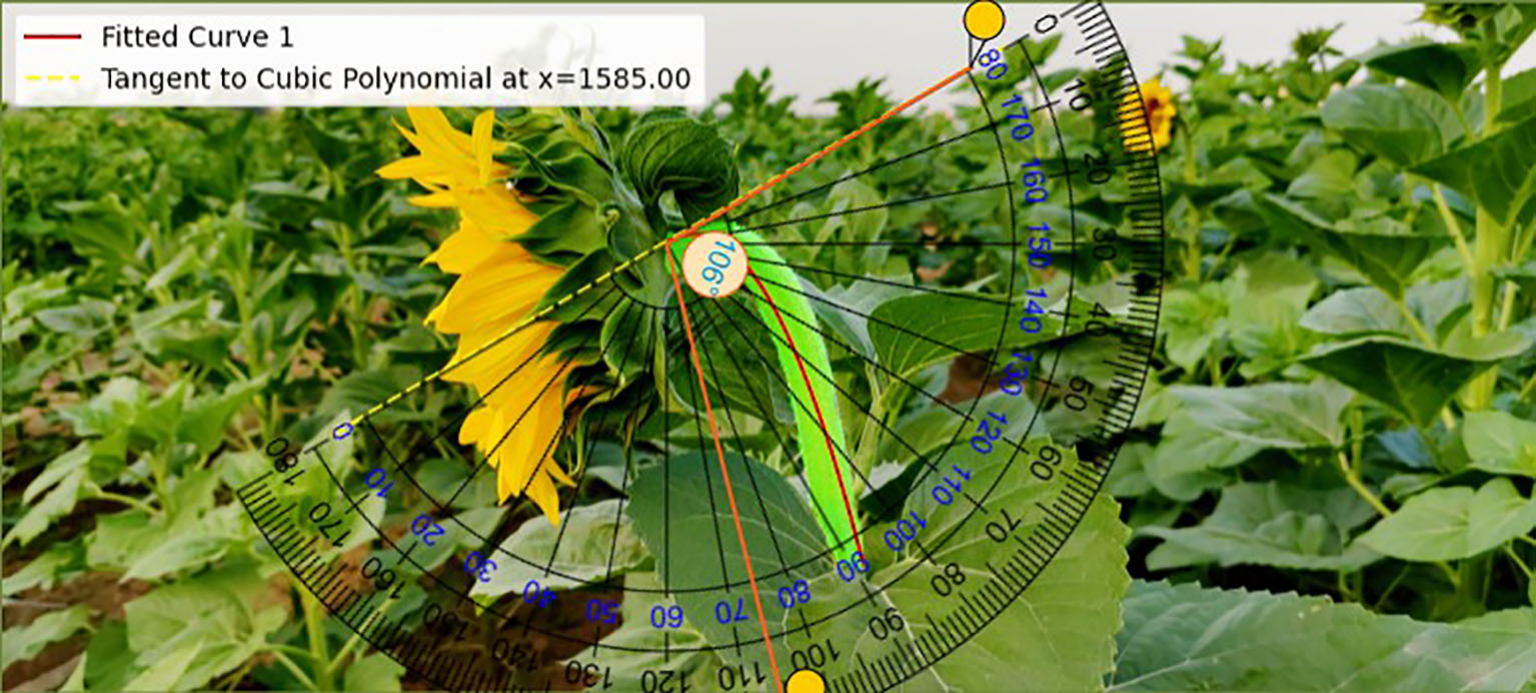
Tilt angle calculation results.

In this study, a set of sample images were used to demonstrate the results comparatively, and the accuracy and feasibility of the method of measuring the inclination angle of the sunflower disk were verified in **Figures 11** and **12** in terms of theoretical calculations and actual measurements, respectively. **Figure 11** demonstrates the tilt angle derived from mathematical fitting and slope calculations and provides theoretical support based on geometric parameters and formulas, which resulted in a calculation of 108.10°. **Figure 12** shows the comparison between the fitting results and the protractor measurements, the measured angle is 106°, which is close to the theoretically calculated value. The experimental results show that the model has good prediction accuracy and generalization ability on the test set. Specifically, the root mean square error of the model is 2.93°, the average absolute error is 2.43°, and the coefficient of determination is 0.94, indicating that the model is able to explain the trend of the data well and the fitting effect is satisfactory. The R² value (0.94) is calculated based on a complete test set of 220 images, without manually removing any outliers.

In order to visualize the performance of the model, scatter plots, error distribution plots, line plots and residual trend plots are used in this paper. Through these graphs, the experimental results are analyzed in detail in terms of prediction accuracy, error distribution, trend fitting ability and residual variation. The experimental results are further illustrated in the following section in conjunction with these graphs.

**Figure 13:**
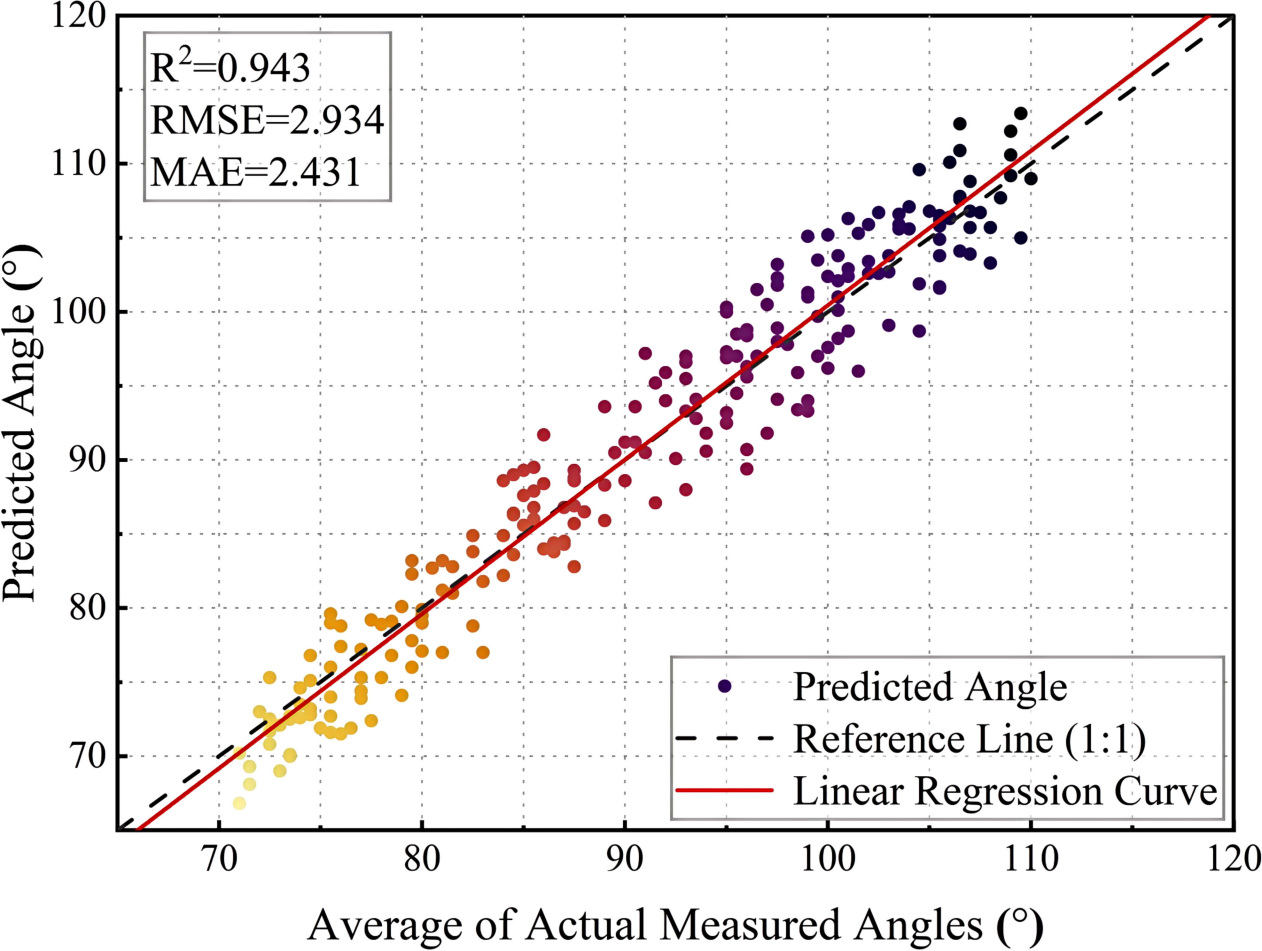
Comparison of projected and actual values.

**Figure 13** illustrates the relationship between the model’s predicted angle and the actual measured angle. The horizontal axis represents the average actual measured angle and the vertical axis represents the predicted angle. The range of angles for the test set is between approximately 70° and 120°. The black dashed line is the 1:1 reference line, which indicates the ideal case where the predicted values are exactly the same as the actual values, and the red solid line is the linear regression fit curve, which is used to show the overall fit of the model. From the scatter distribution, most of the points are close to the reference line, indicating that the model has a high prediction accuracy.

In addition, the model prediction performance for the low angle range ([70°, 80°]) and high angle range ([100°, 110°]) was numerically decomposed. The results show that the fitting performance in these two ranges decreased significantly, as indicated by a significant increase in MAE and RMSE and a significant decrease in R² (see **Table 6** for details). In addition, some prediction points showed significant deviations, which may be caused by the following factors:(1) the complexity of plant growth morphology, such as structural obstruction, flower heads close to the stem, or significant stem curvature, affects the predictive ability of the model; (2) fewer data at extreme angles, which resulted in a weaker fitting ability of the model in these regions, which led to a larger prediction; (3) The amplification effect of the measurement error, where the extreme angles are easily affected by the ambient light, the equipment resolution or the angle calibration error, leading to an increase in the deviation. To address these issues, in the future, the number of extreme angle samples can be increased, the data enhancement method can be optimized, and the angular feature extraction method can be improved to enhance the prediction accuracy of the model in these regions.

**Table 6:** Prediction error decomposition of the model in low-angle and high-angle intervals.

Angle type	Angle range	MAE	RMSE	R²
Low-angle	[70.0, 80.0]	2.18	2.62	0.065
High-angle	[100.0, 110.0]	2.33	2.86	0.085

**Figure 14:**
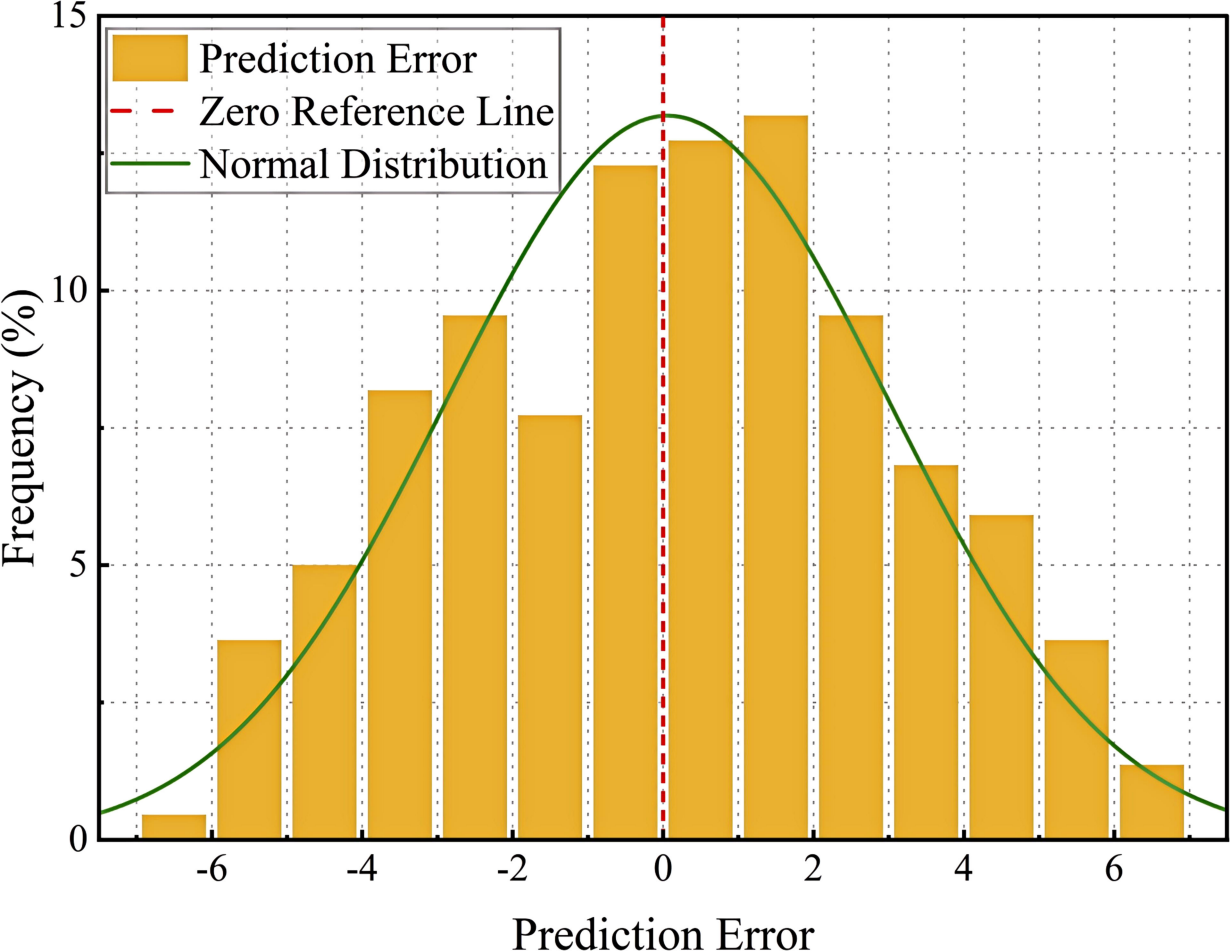
Distribution of prediction errors.

**Figure 14** represents the frequency distribution of the prediction error, with the horizontal axis indicating the value of the prediction error and the vertical axis indicating the frequency percentage of the error. The yellow bar graph shows the distribution of the error, the red dashed line indicates the benchmark of zero error in the ideal state, and the green curve is the normal fitting curve of the error distribution, which is used to reflect the overall error trend. As can be seen from the figure, most of the prediction errors are concentrated around 0, indicating that the overall prediction of the model is stable and the error distribution is close to normal. The standard deviation (2.94) and skewness (0.063) of the errors were calculated to verify whether they were approximately normally distributed. The skewness value is close to 0, indicating that the error distribution is relatively symmetrical and has characteristics of an approximately normal distribution. The standard deviation reflects the degree of fluctuation of the errors. At both ends of the error distribution, there are still a small number of large error points, which may be caused by the following factors: (1) the model fails to adequately learn the features of some samples, resulting in large prediction errors for these samples; (2) measurement errors or data labeling errors, some data points may be affected by human or equipment errors, resulting in large errors; (3) the effect of the concentration of the error, and some of the extreme errors may be due to training imbalance of data distribution, which makes the model’s prediction bias for certain angles increase(). Although the model’s errors are densely distributed around 0, it is still necessary to optimize the outlier handling and reduce the impact of extreme errors on the overall model performance by adjusting the loss function and improving the data processing methods.

**Figure 15:**
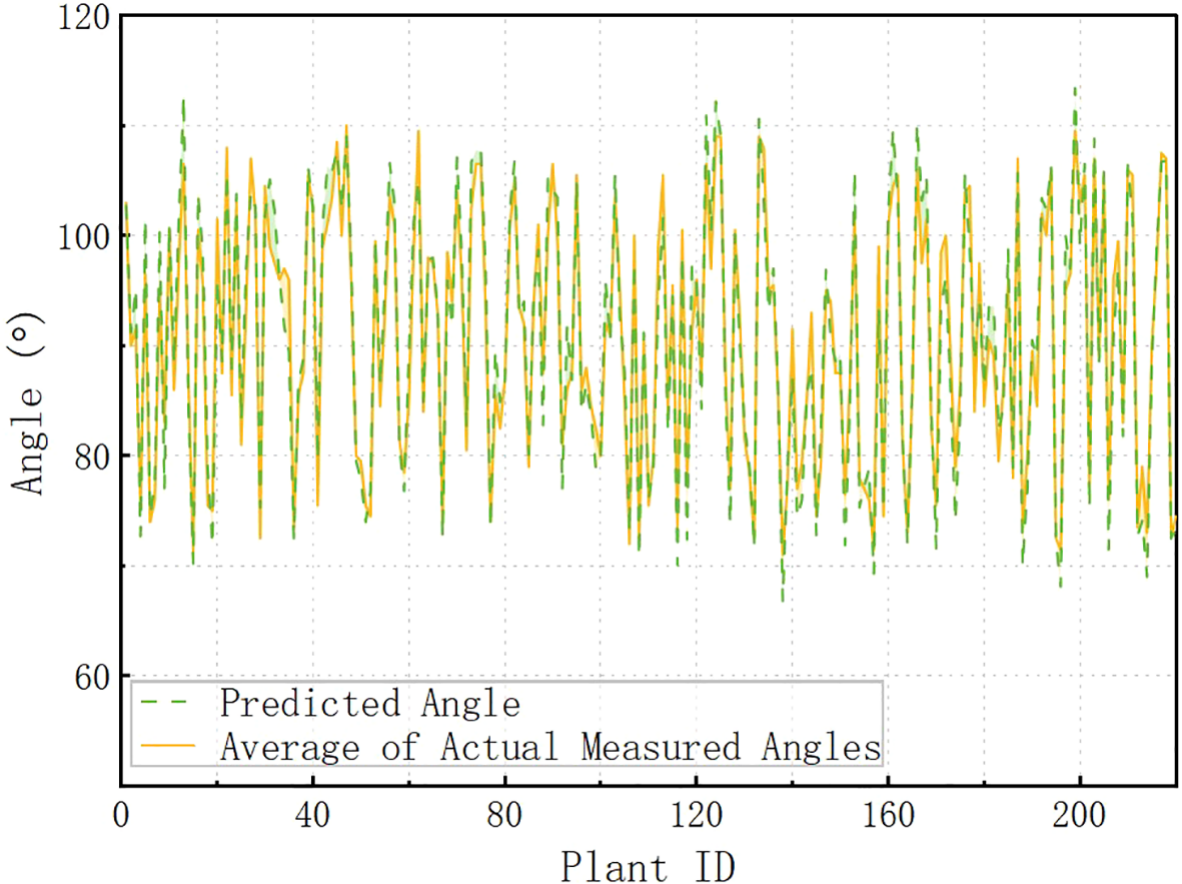
Predicted vs. actual measured angles across Plant IDs.

**Figure 15** illustrates the relationship between the predicted and actual measured angles corresponding to different Plant IDs. The horizontal axis indicates the Plant ID and the vertical axis indicates the angle. The green dashed line indicates the predicted angle, and the yellow solid line indicates the average value of the actual measured angle. From the overall trend, the two trends are basically the same, indicating that the predictive ability of the model is generally good. In the interval of Plant ID 80-120, the predicted angle of some data has obvious deviation from the actual angle, which may be caused by the following factors: (1) the special growth morphology of specific plants, and the plants in this interval may have a large change in the inclination angle, which increases the difficulty of prediction. The “special growth morphology” referred to in this paper mainly refers to structural morphologies that affect angle measurement, such as flower discs close to the stem and curved flower stems, which may cause certain errors in tilt angle measurement. These samples were deliberately retained during collection to reflect morphological diversity. They were not labeled or classified separately during data preprocessing and were used together with other samples during model training and evaluation; (2) the local insufficiency of the training data, and the samples in this interval may be relatively small, which affects the model’s ability of generalization; (3) data anomalies at individual measurement points; this interval may contain certain measurement points that are highly influenced by the environment or equipment, making the prediction error more pronounced. Although the model performs stably in most of the numbered intervals, the error is large in some intervals, and in the future, the data distribution can be optimized to increase the training samples in this interval to reduce the impact of data imbalance on the prediction.

**Figure 16:**
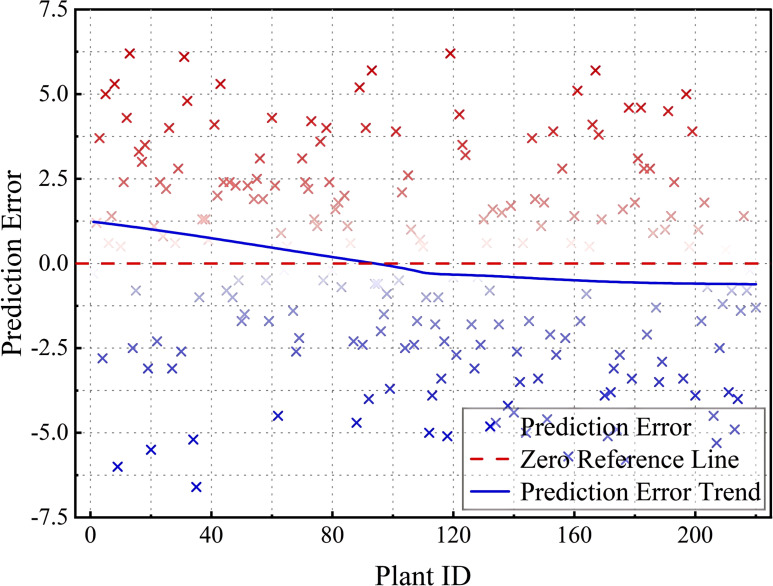
Prediction error distribution across Plant IDs.

**Figure 16** shows the prediction errors corresponding to different Plant IDs, with the horizontal axis indicating the plant number and the vertical axis indicating the prediction error. The red points indicate that the predicted angle is higher than the actual angle, and the blue points indicate that the predicted angle is lower than the actual angle, the red dashed line is the zero error reference line, and the blue trend line shows the trend of the error with Plant ID. Overall, the model error distribution is well-balanced, but some of the error points deviate more (to ±5 or more) in the Plant ID 0–50 and 150–200 intervals. Possible reasons for the large errors in these specific intervals include (1) the greater range of variation in tilt angle of the samples in this interval, making it difficult for the model to accurately predict; (2) uneven distribution of training data, which may lead to insufficient model learning for certain plant numbers, thus affecting the generalization ability; and (3) data quality issues, where some samples may be affected by ambient light, shading, or equipment accuracy, resulting in measurement errors that increase. Although the prediction error of the model is more stable on most Plant IDs, there are still large deviations in some specific intervals. In the future, the data equalization strategy can be used to increase the training data in this interval or adjust the model weights to pay more attention to the regions with larger errors, so as to improve the overall prediction stability.

## 4 Discussion

This study proposes a new method for measuring the tilt angle of sunflower flower heads, based on an optimized YOLO11-seg model and geometric fitting strategy. It demonstrates excellent applicability in terms of measurement accuracy, computational speed, resource consumption, and agricultural applicability. Compared to traditional manual measurement methods, this method enables automatic angle calculation, reduces human visual errors, and effectively avoids plant damage that may result from contact-based measurements. Additionally, the integration of deep learning feature extraction capabilities with geometric modeling achieves a balance between target detection efficiency and spatial structural accuracy.

By comparing with multiple mainstream YOLO series models (such as YOLOv8n-seg, YOLOv9c-seg, YOLO11n-seg, etc.), the final model proposed in this paper achieves significant improvements in both recognition accuracy and stability. Specifically, the final model achieves 0.943 and 0.911 in the two key metrics of Recall and mAP50, respectively, representing improvements of 3.5% and 3.3% compared to the baseline model YOLO11n-seg (Recall 0.908, mAP50 0.878). Additionally, the number of model parameters was reduced from 2.83 million to 2.54 million, and computational complexity was decreased from 10.2 GFLOPs to 9.7 GFLOPs. This significantly reduces resource consumption while maintaining accuracy, thereby enhancing deployment efficiency and practicality.

Given that there are currently no publicly available standard datasets for the task of measuring the tilt angle of sunflower heads, and that related research is still in its exploratory phase, this paper referenced agricultural visual estimation work with certain similarities to the task at hand when evaluating model errors. For example, the A3N model proposed by Wang et al. achieved an average error of 4.8° in fruit grasping direction angle estimation tasks, which can serve as a comparable reference for the results of this study. Although the two studies differ in terms of task objectives, data dimensions, and input modalities, they both address spatial angle estimation problems in agricultural scenarios and thus have certain reference and analogy significance (Wang et al., 2022). In comparison, the method proposed in this paper achieves a measurement accuracy of RMSE = 2.93° and MAE = 2.43°without introducing depth information, utilizing an optimized YOLO11-seg model and geometric fitting strategy, indicating its practical potential and technical feasibility in angle estimation tasks.

Furthermore, to further validate the practical application value of this method, the model calculation results were compared with manual measurement values during the data processing stage. Due to the lack of public reference datasets, an online protractor was used for measurement, and the model error was evaluated using a “manual + benchmark” approach on 220 test images. The experimental results show that the model achieves high consistency with manual measurement results in most samples and maintains stable output when processing flower disk samples at different angles. Compared with manual methods, this method not only effectively reduces human visual errors and improves measurement efficiency but also avoids plant damage that may be caused by contact measurement, making it more practical and deployable.

It should be noted that the performance advantages obtained in this paper may be attributed to factors such as the morphological characteristics of the research subjects, image acquisition strategies, and model structure optimization. In future research, combining three-dimensional sensor information or integrating with multimodal models (such as A3N) to further expand the diversity of training samples in terms of posture and environment may continue to enhance the model’s generalization capabilities and its applicability in complex agricultural environments.

The ablation experiments (T1–T4) further validated the effectiveness of the model structure improvements. The introduction of the CKB module significantly enhances the model’s global modeling capabilities, while the CKBM module improves local context modeling and instance discrimination performance. Although using either module alone can improve performance, the combined use of the CKB and CKBM modules (T4 experiment configuration) yields the best performance across all metrics. The final model demonstrates high-level performance across multiple different structural configurations, indicating its robust and stable performance under various input conditions.

In practical applications, this method has broad potential for widespread adoption. It can be applied to robotic arm path planning in agriculture to assist harvesting robots in accurately identifying and grasping sunflower heads, thereby effectively improving harvesting efficiency and precision. It can also be used for grasping and positioning in tray drying processes to optimize operational workflows (Lammers et al., 2024). Additionally, this method can be extended in the future for automatic measurement of other key structural parameters of plants, such as leaf inclination angle, fruit posture, and branch tilt angle, serving intelligent agriculture scenarios such as crop growth monitoring, yield estimation, and planting management.

Although this study achieved good results, several limitations remain that need to be addressed in future work. First, this method is somewhat dependent on image quality, especially in natural field environments, where complex lighting conditions can cause image degradation, thereby affecting the model’s accuracy in extracting flower disk boundaries and tilt angles. To mitigate this issue, this study introduced an image enhancement strategy based on the Albumentations library during the training phase, through three enhancement techniques: (1) random brightness adjustment (-50% to +50%), (2) random image rotation (-90° to +90°), and (3) addition of Gaussian noise (0% to 20%). These enhancements significantly improved the model’s robustness and generalization ability under varying lighting and pose conditions. However, while image enhancement has played a positive role in improving model performance, the current dataset still lacks sufficient samples of extreme poses or atypical flower disk shapes, leading to slightly higher prediction errors on such samples. Additionally, the enhancement strategies primarily rely on pixel-level perturbations and have not yet adequately addressed systematic changes that may occur in diverse natural environments, such as severe occlusions or complex background interference. Future research could further incorporate multi-source image acquisition, cross-seasonal/cross-regional data augmentation, or combine style transfer techniques to simulate more diverse natural variation scenarios, thereby enhancing the model’s stability and adaptability in real-world agricultural applications.

Secondly, although multiple measurements and averaging strategies were employed in the experiment to reduce human annotation errors, human measurements themselves still exhibit a certain degree of bias. Furthermore, the method proposed in this paper is based solely on two-dimensional image input, which carries the risk of projection angle errors. Future research could consider incorporating depth cameras to collect three-dimensional information, enabling more accurate modeling of spatial structures (Moaven et al., 2024). It should be noted that while this paper proposes the future direction of three-dimensional information fusion, no actual measurements or simulations of 3D models have been conducted at this stage, and the related prospects remain theoretical feasibility analyses.

Meanwhile, since the model training data comes from mature stages and natural field environments, its generalization ability under extreme weather conditions, severe occlusion, or highly variable plant morphology still needs further validation (Gao et al., 2020). Future research could enhance the model’s generalization performance by expanding the sample repository to include different growth stages and diverse plant morphologies; introduce multimodal sensors (e.g., infrared, depth information) to complement the limitations of single visual information (Chen et al., 2023); and explore light compensation strategies such as integrating low-power LED supplemental lighting devices to improve robustness in low-light environments. In terms of structural optimization, further refinement of geometric fitting methods and deep neural network architectures can improve adaptability and prediction accuracy under extreme conditions (Zhao et al., 2024).

Finally, integrating this method with robotic systems enables real-time tilt angle sensing and dynamic control during operations (Dumberger et al., 2020), providing technical support for agricultural automation. By converting this method into an application product and achieving large-scale deployment, it is expected to drive the development of smart agriculture toward higher precision, efficiency, and robustness, thereby facilitating the modernization of agricultural production methods.

## 5 Conclusion

As agricultural modernization continues to advance, traditional contact-based and manual measurement methods are increasingly revealing limitations in terms of efficiency, accuracy, and application safety. To meet the demand for efficient, non-destructive, and automated operations in agricultural production, developing an accurate and stable method for measuring the tilt angle of sunflower flower heads is of great significance. Accurately identifying the tilt angle of the flower head is not only a key foundation for path planning in intelligent harvesting systems but also directly impacts the precision and efficiency of subsequent operations. Especially under complex lighting conditions and diverse plant morphologies in natural fields, the robustness of the detection method is the core guarantee for the stable operation of the system.

This study proposes a non-contact method for estimating the tilt angle of sunflower heads by combining an optimized deep learning model with a geometric fitting strategy. The method enhances the model’s ability to represent spatial features by introducing CKB and CKBM modules, which improve key feature representation and instance discrimination performance in the object detection process from the perspectives of global structural modeling and local context awareness, respectively. Additionally, a lightweight network structure reduces computational complexity, and multiple image enhancement strategies significantly improve the model’s robustness and generalization capabilities in complex environments. Experiments demonstrate that the model achieves an angle estimation accuracy of MAE = 2.43° and RMSE = 2.93° on 220 natural field images. In terms of mAP50 and Recall, the model achieves 0.911 and 0.943, respectively, representing improvements of 3.3% and 3.5% over YOLO11n-seg, while also reducing computational resource consumption and outperforming mainstream models such as YOLOv8n-seg and YOLOv9c-seg. Ablation experiments further validate the role of each structural improvement module in enhancing overall performance, confirming the effectiveness and rationality of the model optimization strategy. In addition, this paper also conducted a comparative experiment between model prediction and manual measurement during the data collection stage. The results show that this method exhibits consistency and stability under different flower disk inclination angles, demonstrating good practical value. Compared with traditional methods, this research plan can significantly reduce human error and the risk of plants damage, improve measurement efficiency, and facilitate deployment in actual agricultural operation scenarios.

Despite achieving good performance, the study still has some limitations, such as the need to enhance adaptability to flower heads with special growth angles, and projection errors in occluded environments when using a two-dimensional input method. Future research will focus on further developing multi-modal data fusion and three-dimensional perception modeling data expansion. The proposed method has good portability and can be integrated into agricultural robot systems to provide technical support for key intelligent agricultural tasks such as sunflower crop phenotyping monitoring, precise harvesting, and structural recognition.

## Data Availability

The raw data supporting the conclusions of this article will be made available by the authors, without undue reservation.
